# Supplementary material for: Ferromagnetic Fe-TiO2 spin catalysts for enhanced ammonia electrosynthesis
Source: Nat Commun. 2025 Jan 28;16:1129. doi: 10.1038/s41467-025-56566-7 (PMC11775347; doi:10.1038/s41467-025-56566-7)
Supplement: Supplementary file 1 — Supplementary Information [file 41467_2025_56566_MOESM1_ESM.pdf]

Supplementary information

Ferromagnetic Fe-TiO_2 spin catalysts for enhanced ammonia electrosynthesis

Jingnan Wang [1,2‡], Kaiheng Zhao [3‡], Yongbin Yao [4], Fan Xue [4], Fei Lu [5], Wensheng Yan [6], Fangli Yuan [7], and Xi Wang [2*]

[1] Institute of Molecular Engineering Plus, College of Chemistry, Fuzhou University, Fuzhou 350108, China.

[2] State Key Laboratory of Heavy Oil Processing, China University of Petroleum, Beijing, 102249, P. R. China

[3] Key Laboratory of Photochemistry, Institute of Chemistry, Chinese Academy of Sciences, Beijing 100190, P. R. China

[4] Key Laboratory of Luminescence and Optical Information, Ministry of Education, School of Physical Science and Engineering, Beijing Jiaotong University, Beijing 100044, P. R. China.

[5] College of Physical Science and Technology, Yangzhou University, Yangzhou 225002, P. R. China

[6] National Synchrotron Radiation Laboratory, University of Science and Technology of China, Hefei, Anhui, 230026 P. R. China

[7] State Key Laboratory of Mesoscience and Engineering, Institute of Process Engineering, Chinese Academy of Sciences (CAS), Beijing 100190, P. R. China

‡These authors contributed equally.

Email: wangxicas@gmail.com (or xiwang@bjtu.edu.cn)

This file includes:

Supplementary Figures 1-57.

Supplementary Tables 1-8.

Supplementary References.

Supplementary Figures and Tables

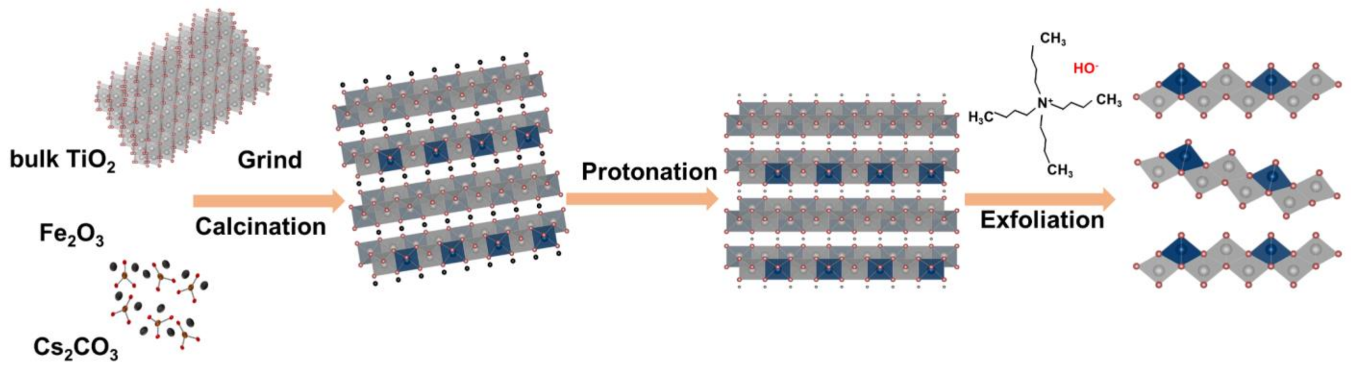

Supplementary Fig. 1 | Schematic diagram of Fe−TiO_2 nanosheets synthetic procedure: Fe (blue), Ti(gray), O (red).

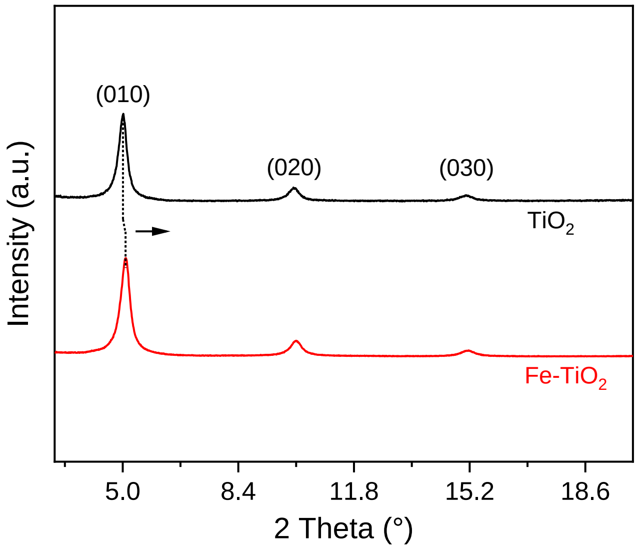

Supplementary Fig. 2 | XRD Spectra of TiO_2 and Fe-TiO_2. Source data are provided as a Source Data file.

Characteristic peaks at 2θ = 5°, 10°, and 15° correspond to (010), (020), and (030) crystal planes of TiO_2, respectively [1].

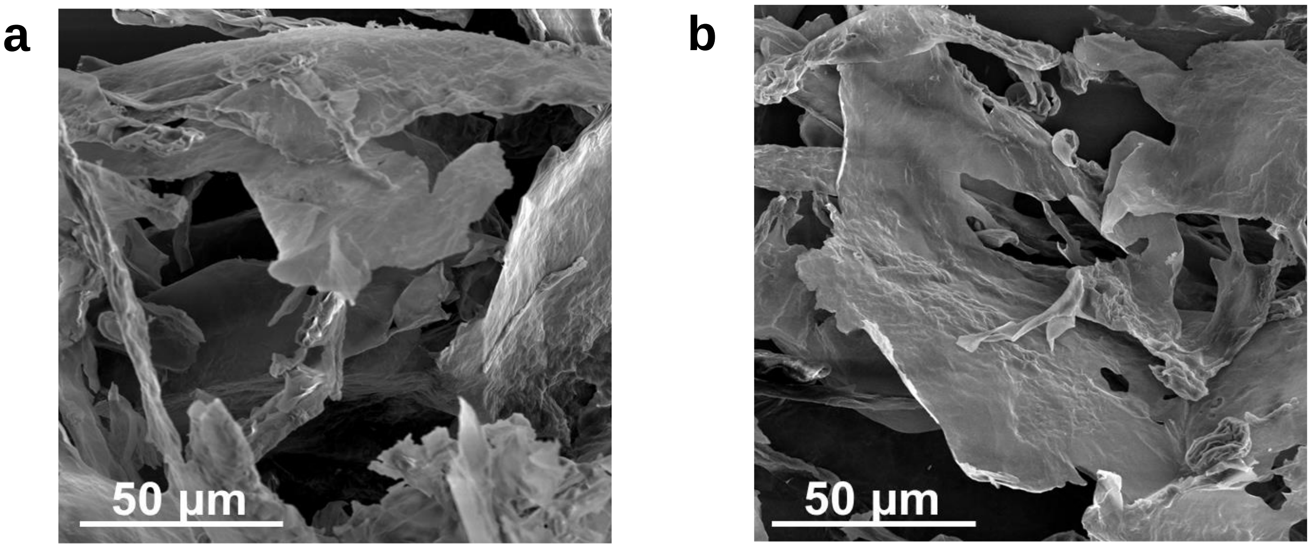

Supplementary Fig. 3 | SEM Images of TiO_2 (**a**) and Fe-TiO_2 (**b**).

The catalyst's ultra-thin sheet-like structure can be easily observed in the SEM images.

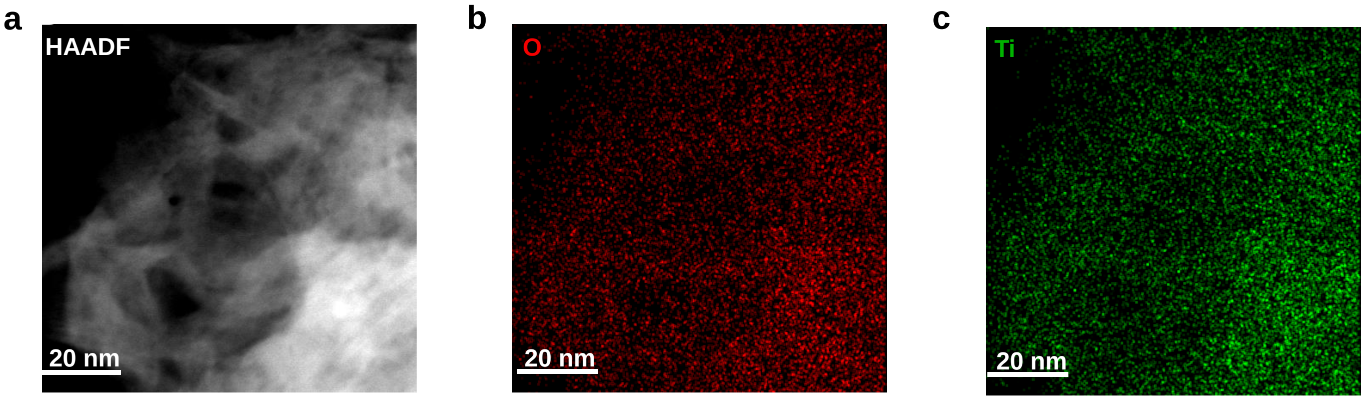

Supplementary Fig. 4 | HRTEM Images of TiO_2 and Corresponding HAADF Images (**a**) and EDS Mapping (**b**, **c**).

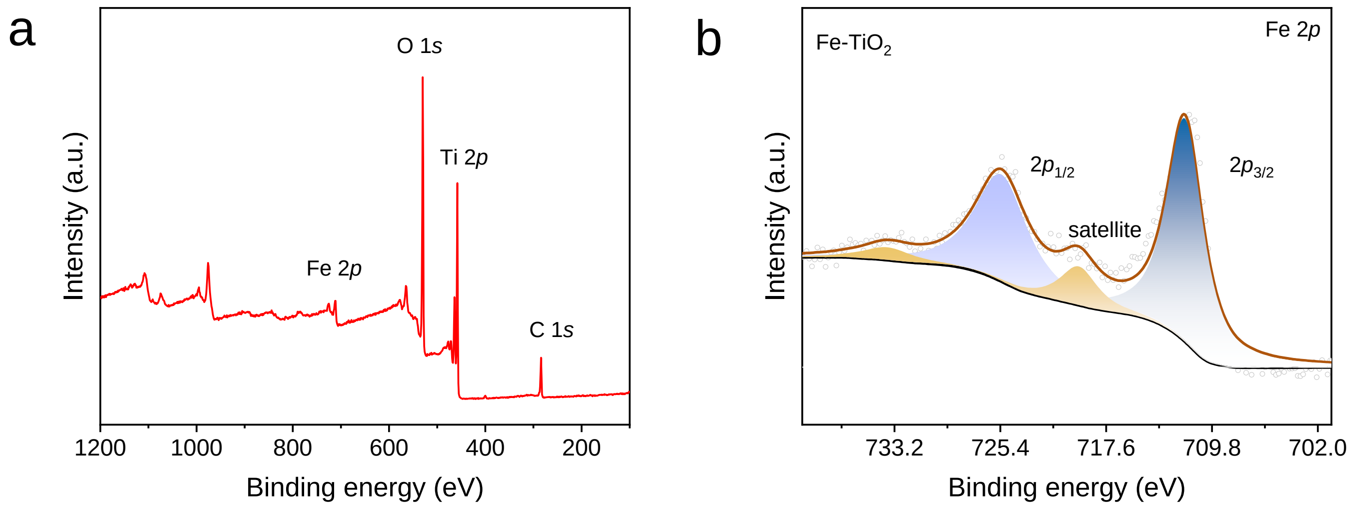

Supplementary Fig. 5 | XPS Full Spectrum (**a**) and Fe 2p XPS High-Resolution Spectrum (**b**) of Fe-TiO_2. Source data are provided as a Source Data file.

The 13.5 eV splitting of the 2*p* orbitals in Fe-TiO_2 due to spin-orbit coupling is smaller than the 13.7 eV in Fe_2O_3, confirming the unsaturated coordination of Fe in Fe-TiO_2.

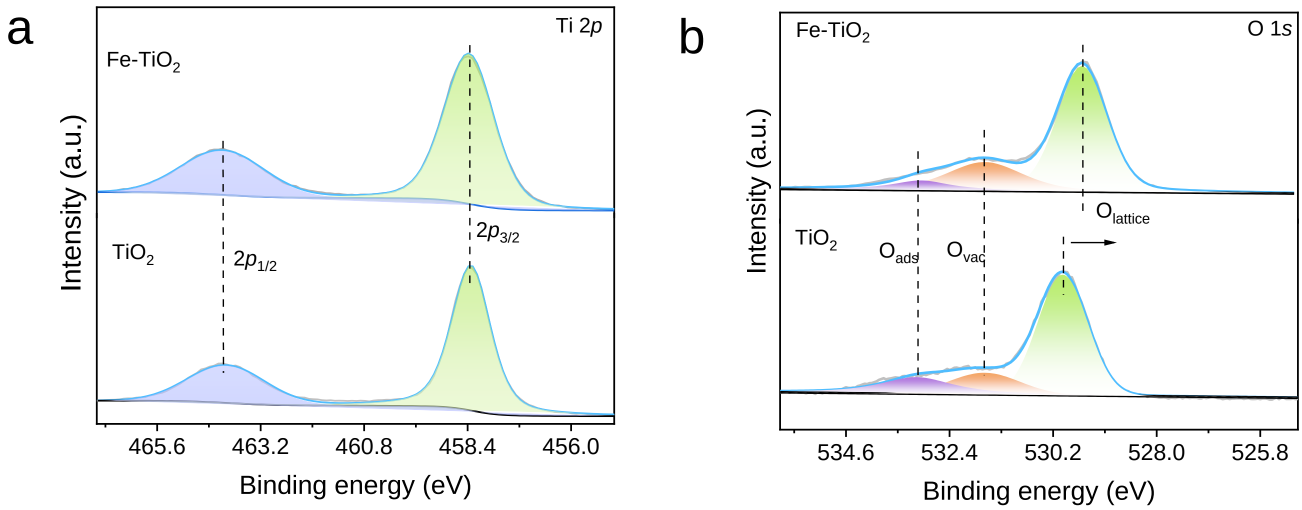

Supplementary Fig. 6 | Ti 2*p* (**a**) and O 1*s* (**b**) XPS high-resolution spectra of Fe-TiO_2 and TiO_2. Source data are provided as a Source Data file.

Due to the incorporation of Fe, some electrons have transferred from Fe to TiO_2, resulting in a shift of the XPS characteristic peaks of O towards lower binding energies.

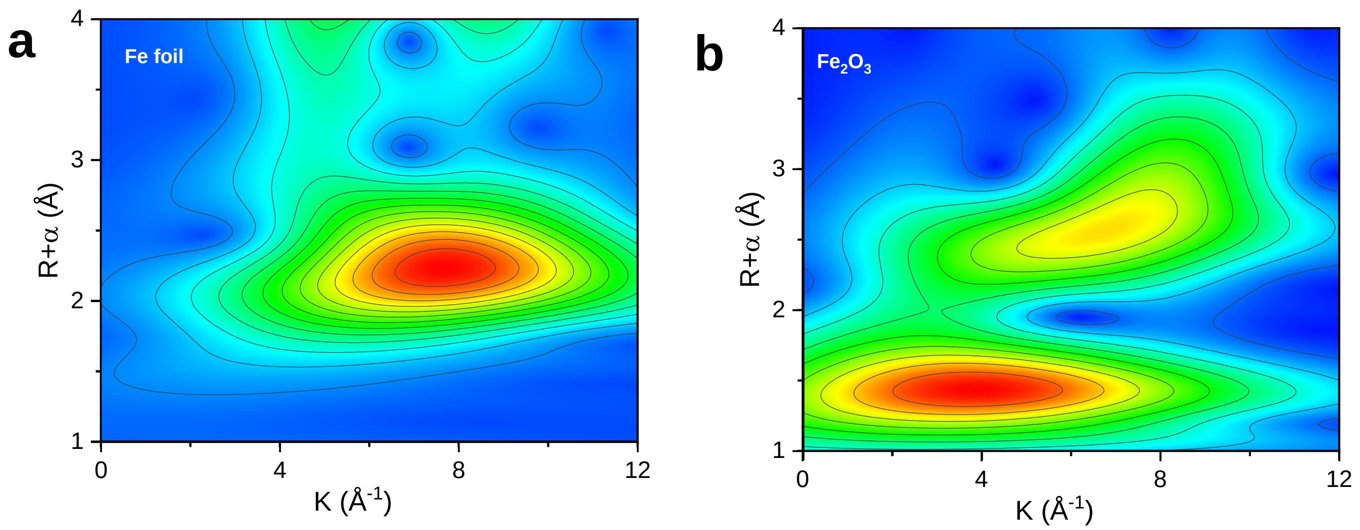

Supplementary Fig. 7 | Wavelet transform EXAFS of the k^2-weighted k space of Fe foil (**a**) and Fe_2O_3 (**b**).

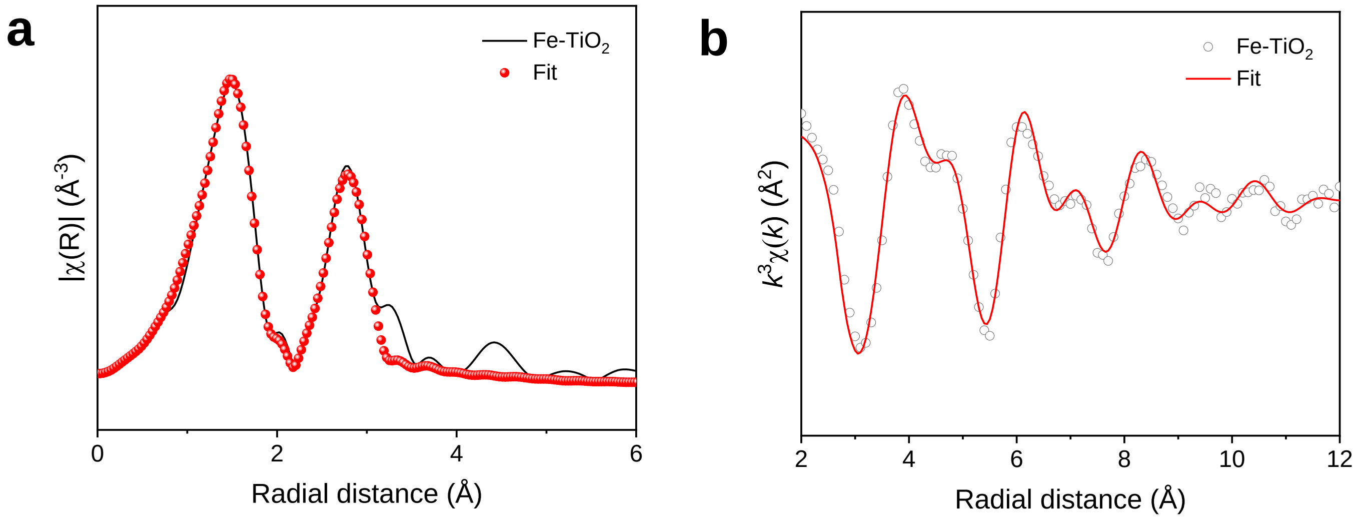

Supplementary Fig. 8 | (a), Fourier transform of EXAFS fitting curve of Fe-TiO_2. **(b)**, The k-space EXAFS fitting curve of Fe-TiO_2. Source data are provided as a Source Data file.

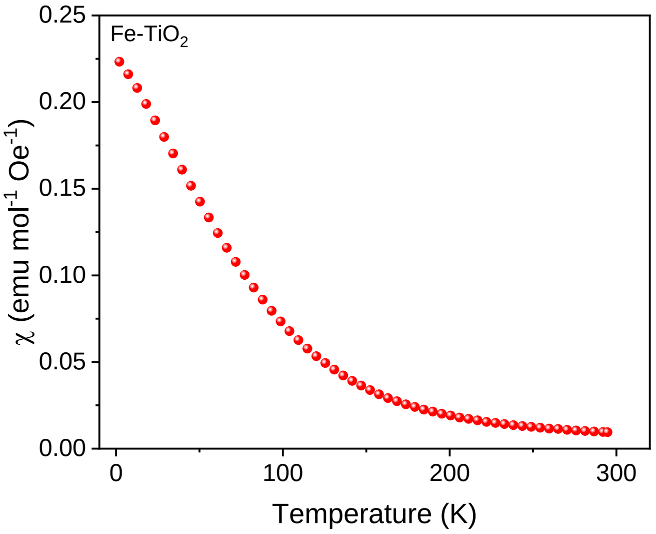

Supplementary Fig. 9 | The temperature dependence of magnetization for Fe-TiO_2. Source data are provided as a Source Data file.

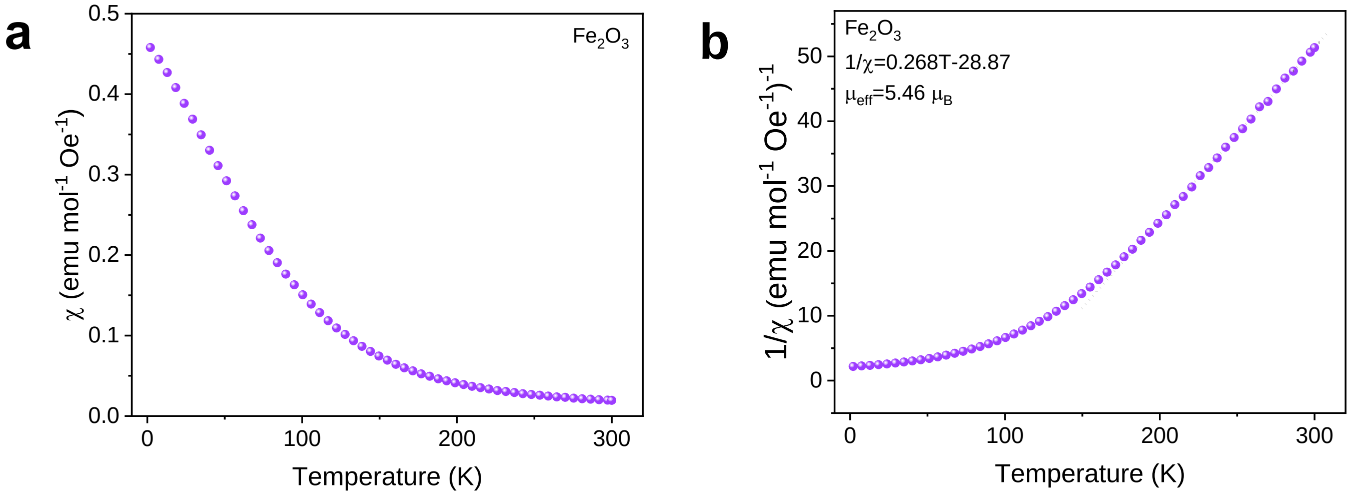

Supplementary Fig. 10 |(a) The temperature dependence of magnetization for Fe_2O_3. And corresponding temperature-dependent inverse susceptibility $1/\chi$ for Fe_2O_3 catalyst at 1000 Oe (**b**). Source data are provided as a Source Data file.

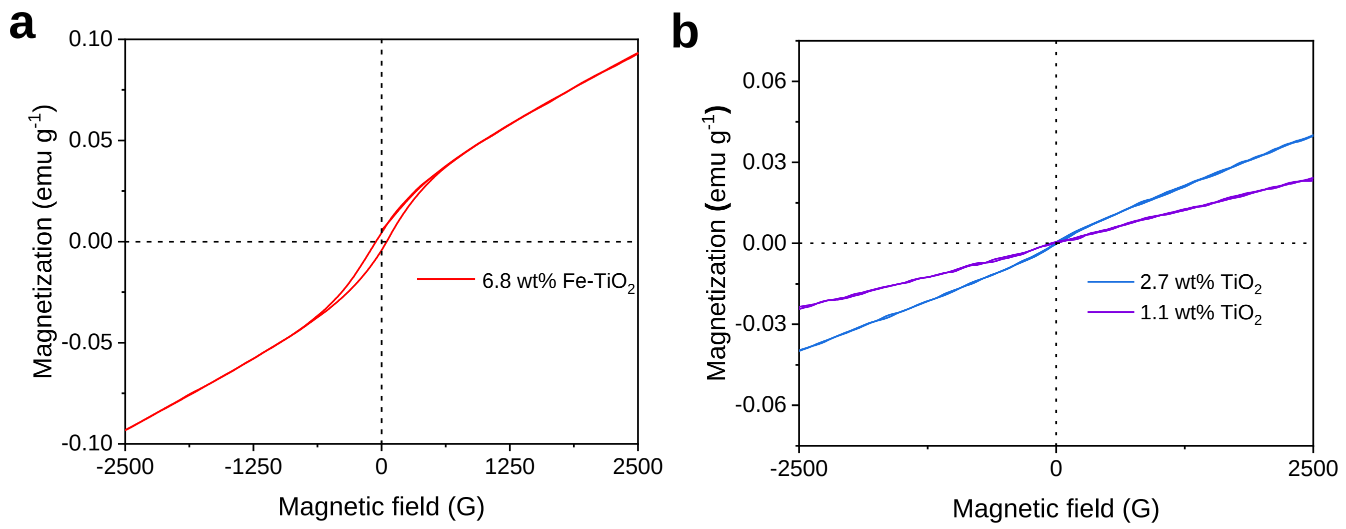

Supplementary Fig. 11 | The spin state diagram of Fe-TiO_2 catalyst. (**a**) 6.8 wt% asnd (**b**) 2.7 wt% and 1.1 wt%. Source data are provided as a Source Data file.

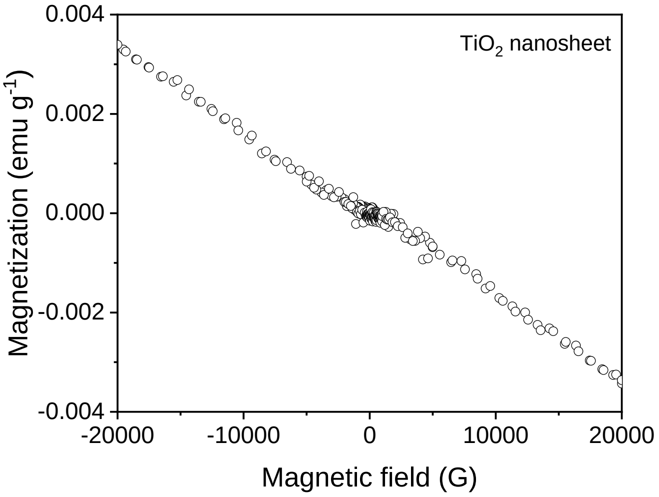

Supplementary Fig. 12 | Magnetization curves (M-H) of TiO_2 catalyst powders at room temperature (300 K). Source data are provided as a Source Data file.

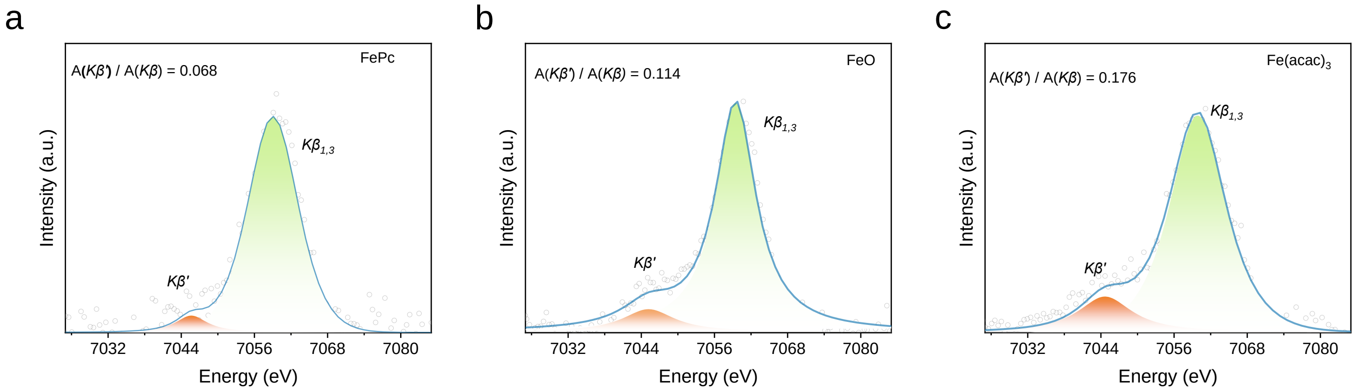

Supplementary Fig. 13 | The Fe kβ XES and corresponding peak area fitting of (**a**) FePc, (**b**) FeO, (**c**) $Fe(acac)_3$. Source data are provided as a Source Data file.

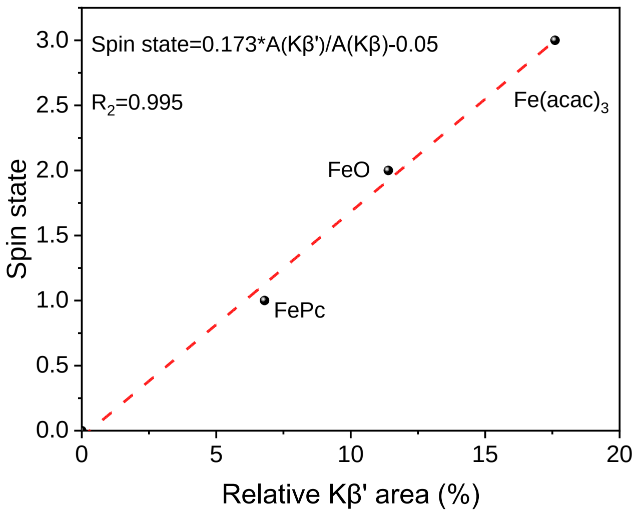

Supplementary Fig. 14 | The relationship between the relative area of the Kβ' peak in the standard sample and the corresponding spin states, along with the linear fitting curve. Source data are provided as a Source Data file.

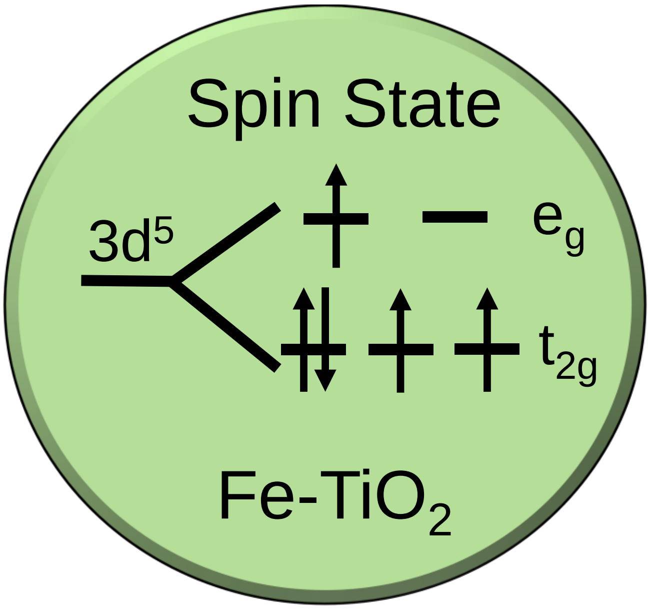

Supplementary Fig. 15 | The spin state diagram of Fe-TiO_2 catalyst.

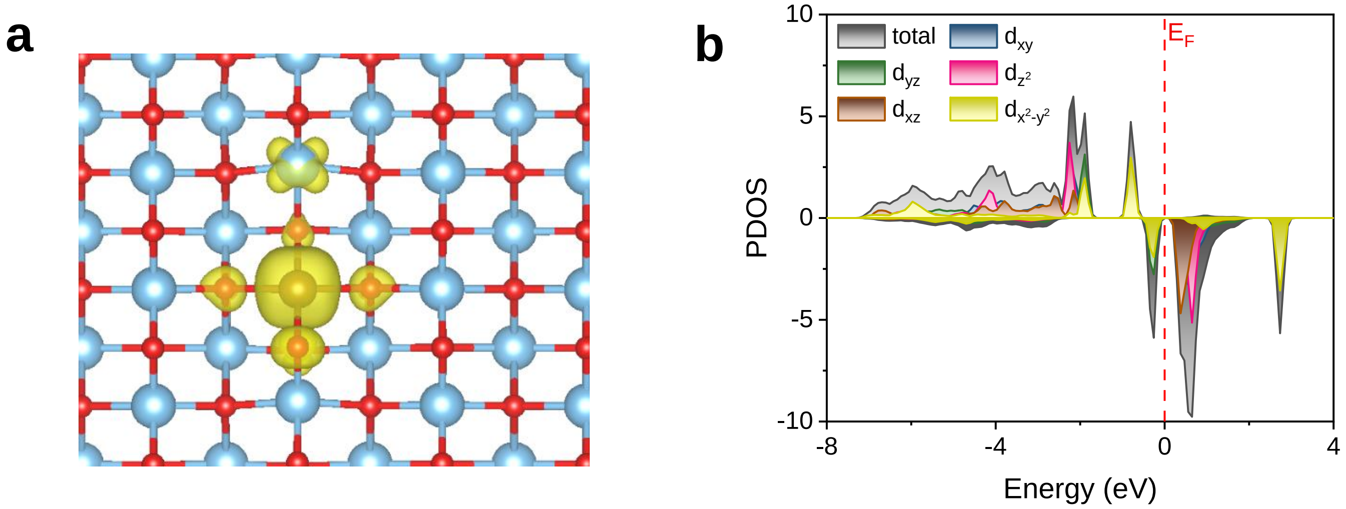

Supplementary Fig. 16 | Spin charge density (**a**) and Projected density of states (**b**) for Fe in Fe-TiO_2.

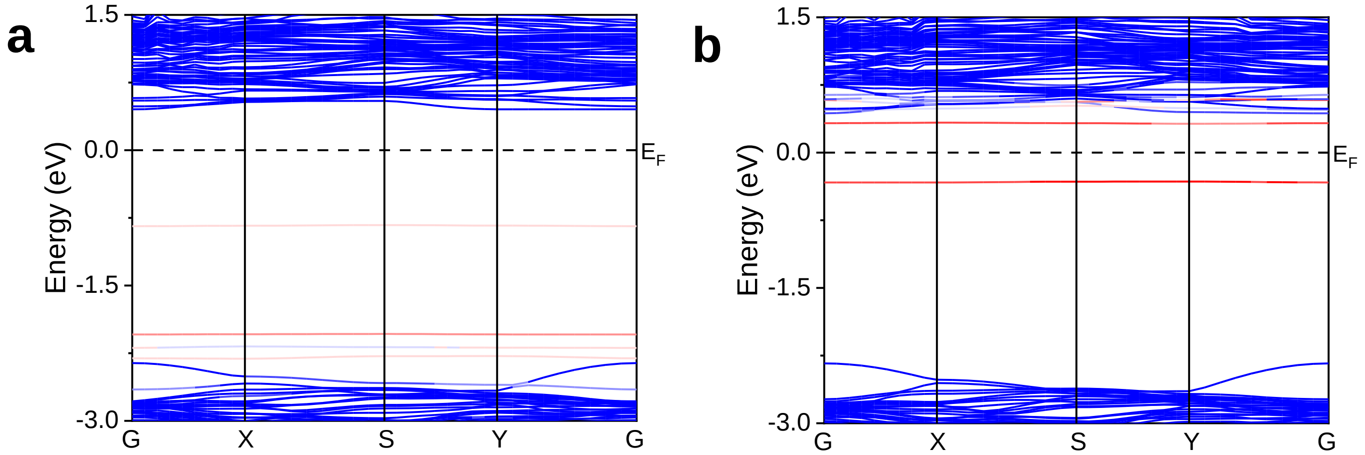

Supplementary Fig. 17| Spin-up (**a**) and spin-down (**b**) band structures for Fe-TiO_2. Red indicates the contribution from Fe atoms, blue denotes the contribution from TiO_2. Source data are provided as a Source Data file.

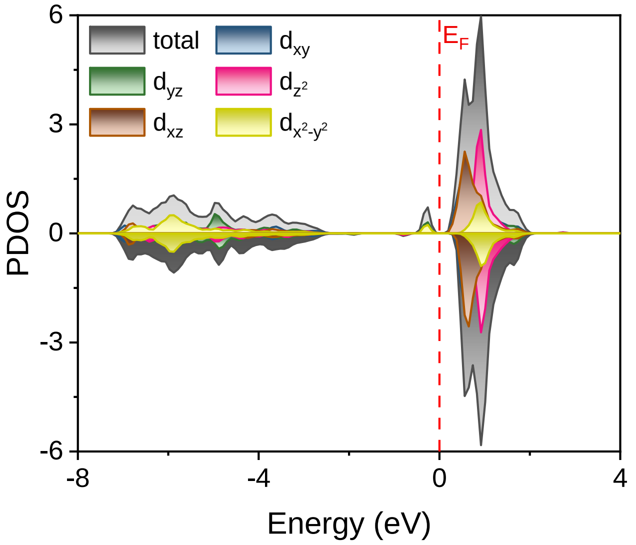

Supplementary Fig. 18 | Projected density of states for Ti in Fe-TiO_2. Source data are provided as a Source Data file.

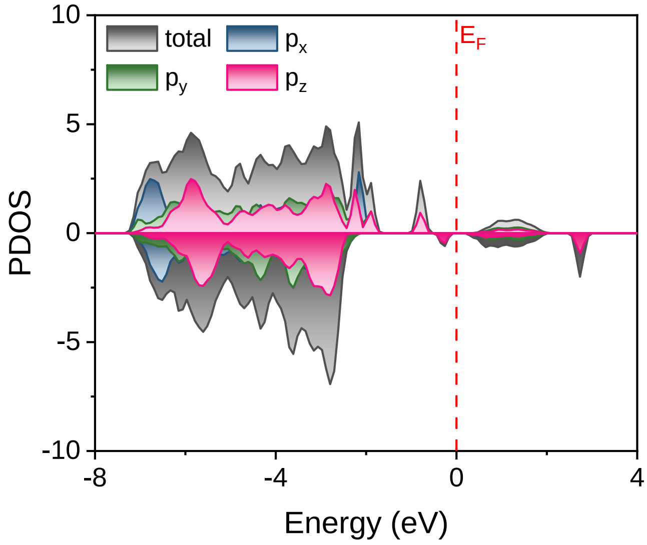

Supplementary Fig. 19 |Projected density of states nearest-neighbor O in Fe-TiO_2. Source data are provided as a Source Data file.

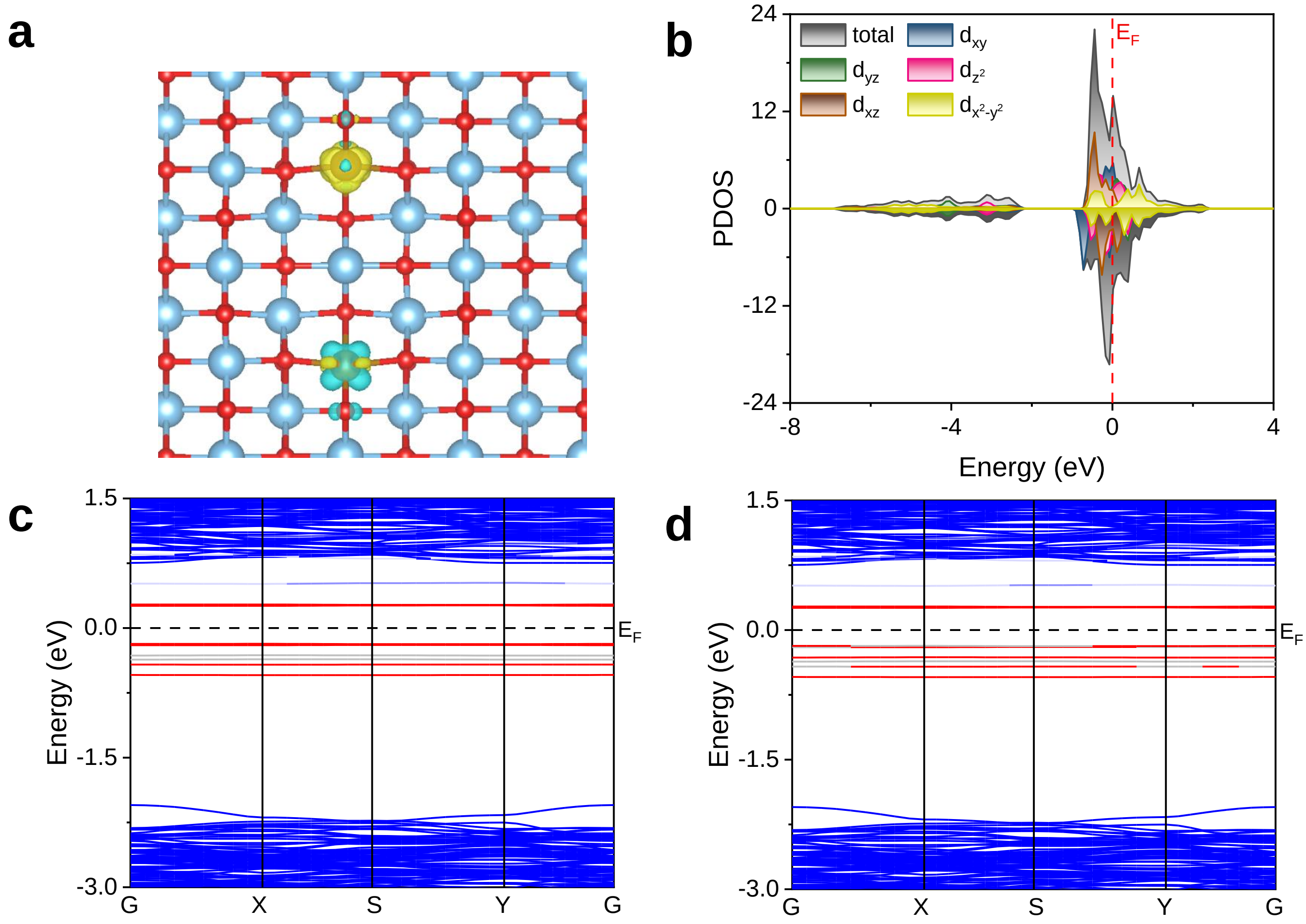

Supplementary Fig. 20 | The electronic structure of Fe-TiO_2 without spin alignment. (**a**) Spin charge density, (**b**) Projected density of states for Fe, (**c**, **d**) Spin-up and spin-down band structures for Fe-TiO_2 without spin alignment. Red indicates the contribution from Fe atoms, blue denotes the contribution from TiO_2. Source data are provided as a Source Data file.

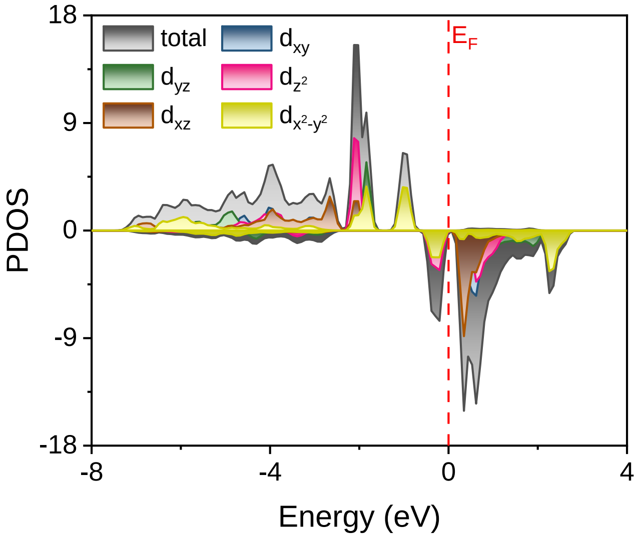

Supplementary Fig. 21 | Projected density of states for Fe in ferromagnetic Fe-TiO_2 with spin alignment. Source data are provided as a Source Data file.

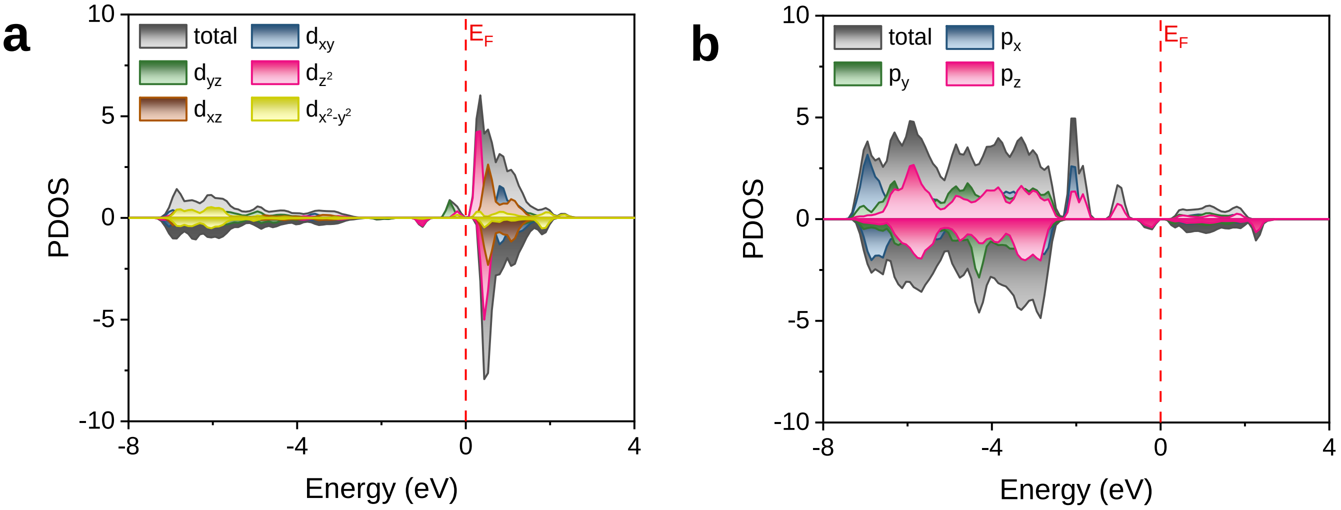

Supplementary Fig. 22 | Projected density of states for (**a**) Ti and (**b**) nearest-neighbor O in Fe-TiO_2 with spin alignment. Source data are provided as a Source Data file.

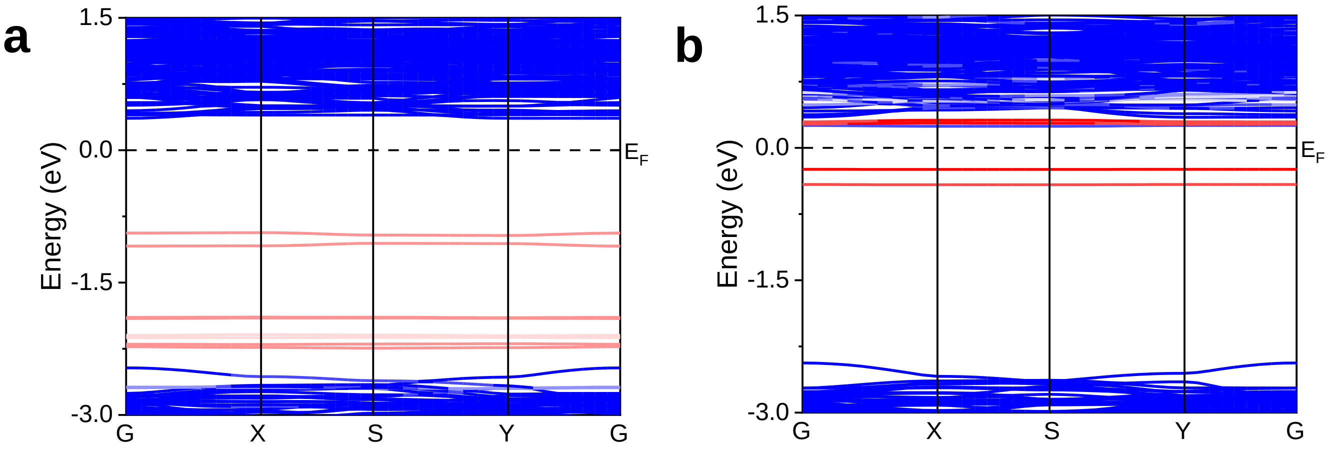

Supplementary Fig. 23 | Spin-up (**a**) and spin-down (**b**) band structures for Fe-TiO_2 with spin alignment. Red indicates the contribution from Fe atoms, blue denotes the contribution from TiO_2. Source data are provided as a Source Data file.

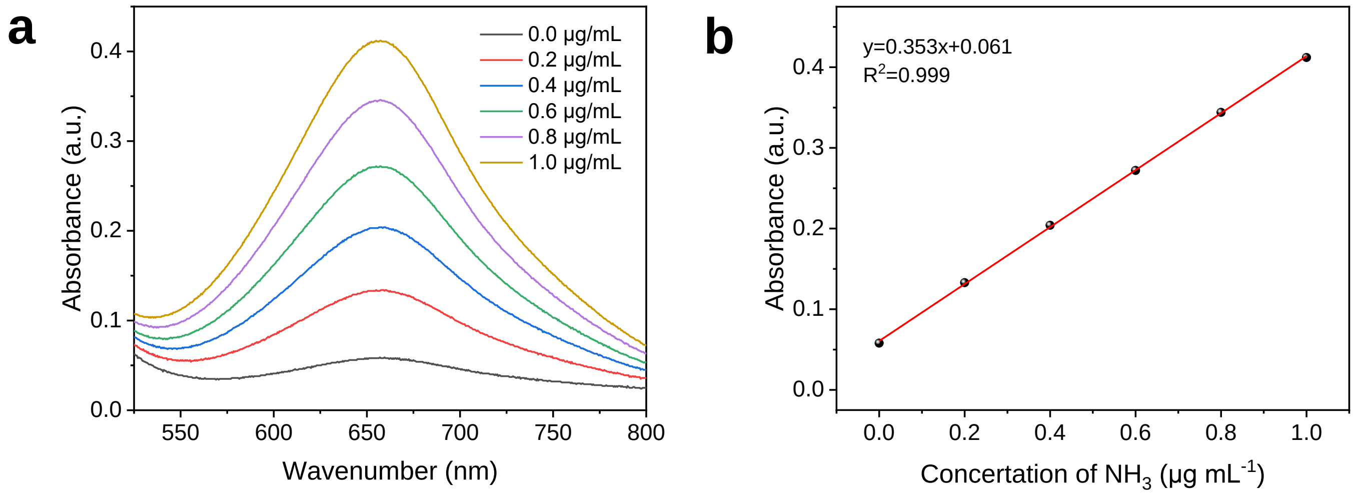

Supplementary Fig. 24 | Diluting with a standard NH_4^+ solution to obtain known NH_3 concentrations, the corresponding UV-visible absorption curves were obtained using the indophenol blue method (**a**). By selecting the absorbance at 655 nm and fitting, the linear relationship between NH_4^+ concentration and absorbance was determined (**b**). Source data are provided as a Source Data file.

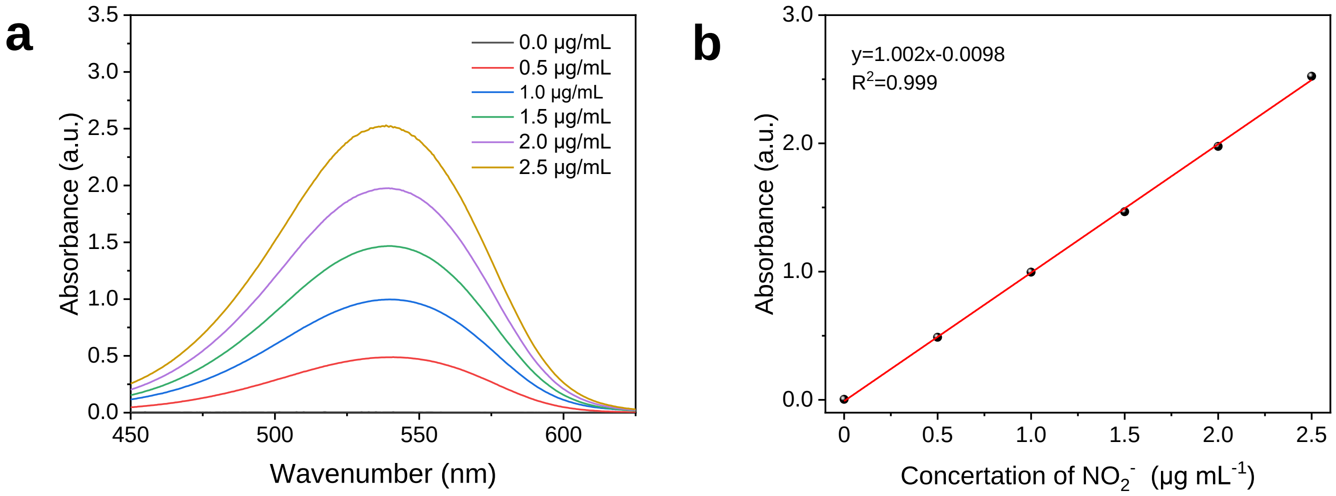

Supplementary Fig. 25 | Using a known concentration of KNO_2 solution, the corresponding UV-visible absorption curves were obtained with the Griess method (**a**). By selecting the absorbance at 540 nm and fitting, the linear relationship between NO_2^- concentration and absorbance was determined (**b**). Source data are provided as a Source Data file.

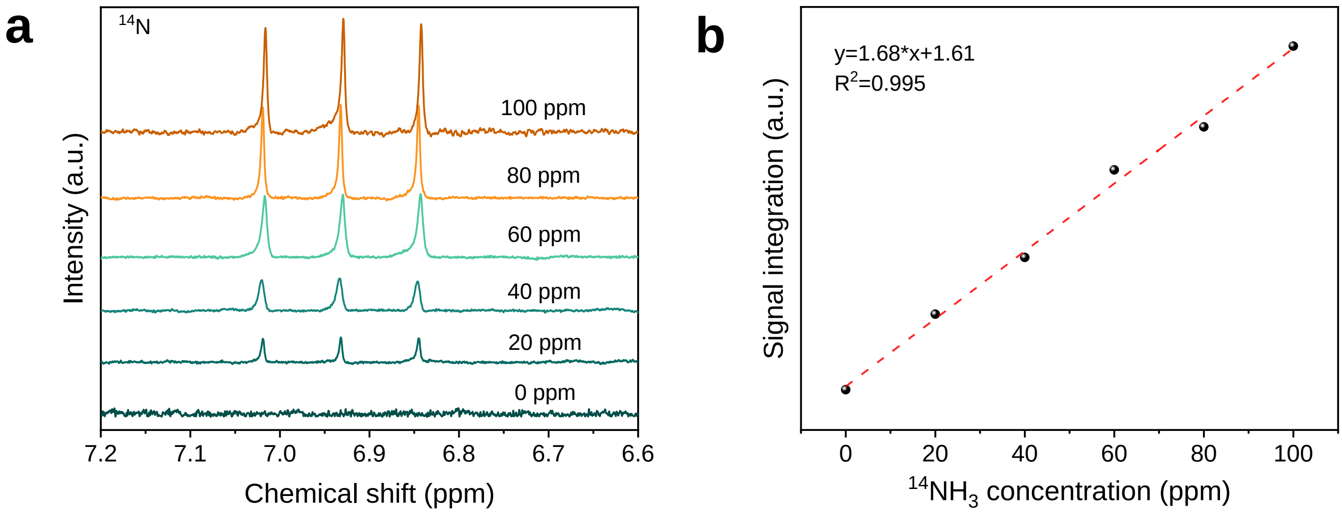

Supplementary Fig. 26 | Dilution of known concentration of NH_4^+ standard solution and corresponding ^{1}H NMR Spectrum (**a**) and linear relationship between NH_3 concentration and peak area (**b**). Source data are provided as a Source Data file.

The ^{1}H NMR spectrum of $^{14}NH_4^+$ shows a triplet peak, while the 1H NMR spectrum of $^{15}NH_4^+$ exhibits a doublet peak.

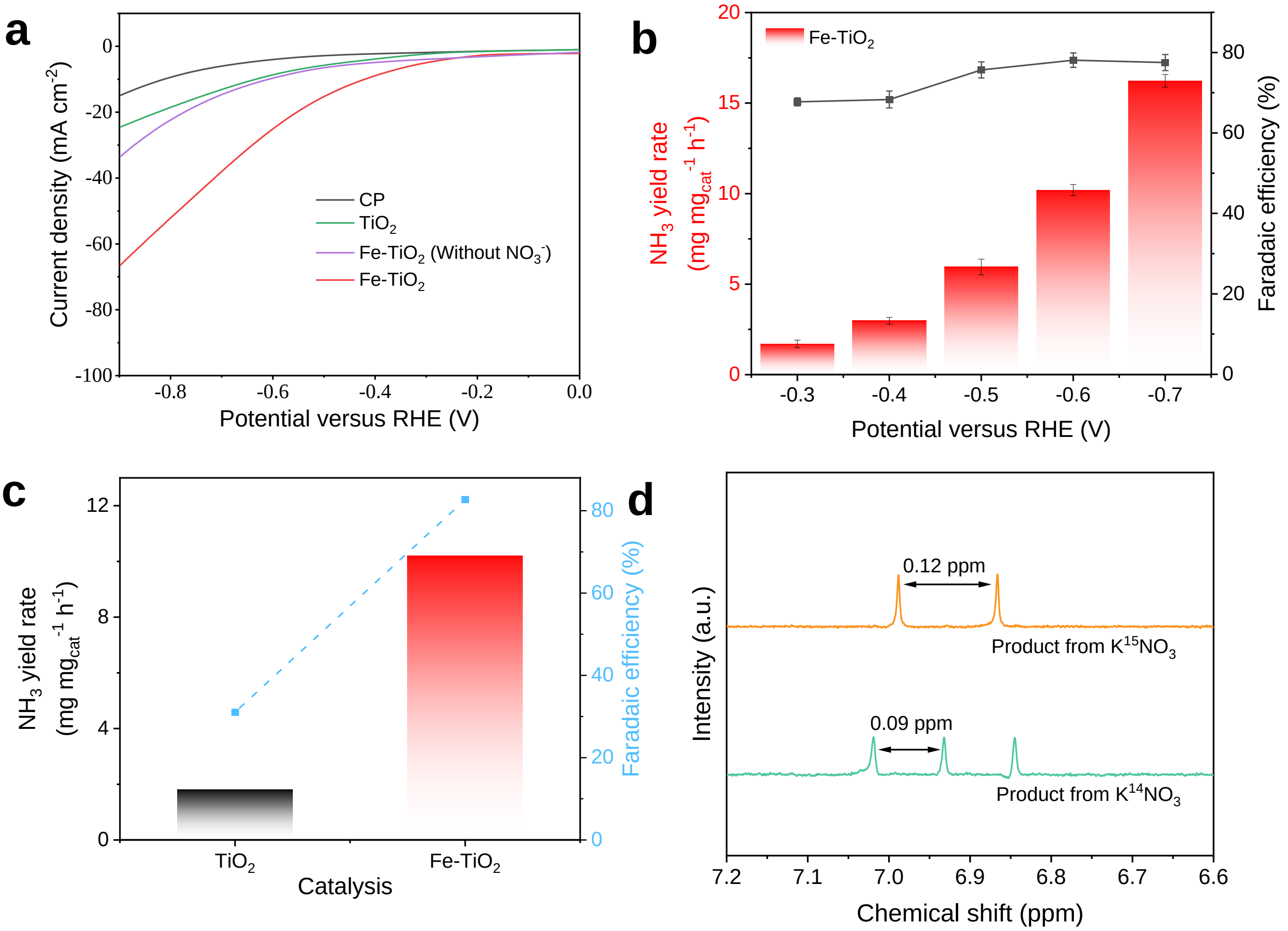

Supplementary Fig. 27 | Electrocatalytic nitrate reduction for ammonia synthesis performance of TiO_2 and Fe-TiO_2 catalysts in Ar-saturated 0.1 M KOH containing 0.1 M KNO_3 electrolyte. (**a**) The linear sweep voltammetry (LSV) curves of Carbon Paper, TiO_2 and Fe-TiO_2 catalysts. (**b**) NH_3 yield and FE_{NH3} of Fe-TiO_2 catalyst at various potentials. (**c**) NH_3 yield and FE_{NH3} of TiO_2 and Fe TiO_2 catalysts at -0.6V vs. RHE. (**d**) ^1H NMR spectra of electrolytes after electrocatalytic using $^{14}NO_3^-$ and $^{15}NO_3^-$ as a N source. All electrochemical data shown in the figures are not iR-corrected. Source data are provided as a Source Data file.

Fe-TiO_2 exhibited higher current density compared to carbon paper (CP) and TiO_2. Additionally, the inclusion of KNO_3 led to a significant enhancement in current density compared to its absence.

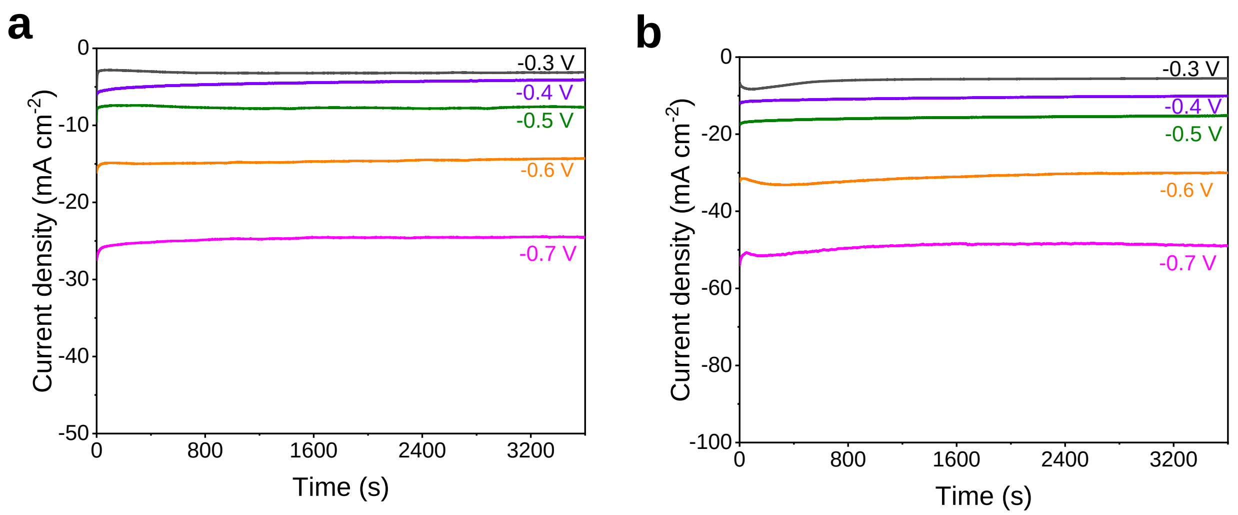

Supplementary Fig. 28 | I-t curve for TiO_2 (**a**) and Fe-TiO_2 (**b**) at different potentials. All electrochemical data shown in the figures are not iR-corrected. Source data are provided as a Source Data file.

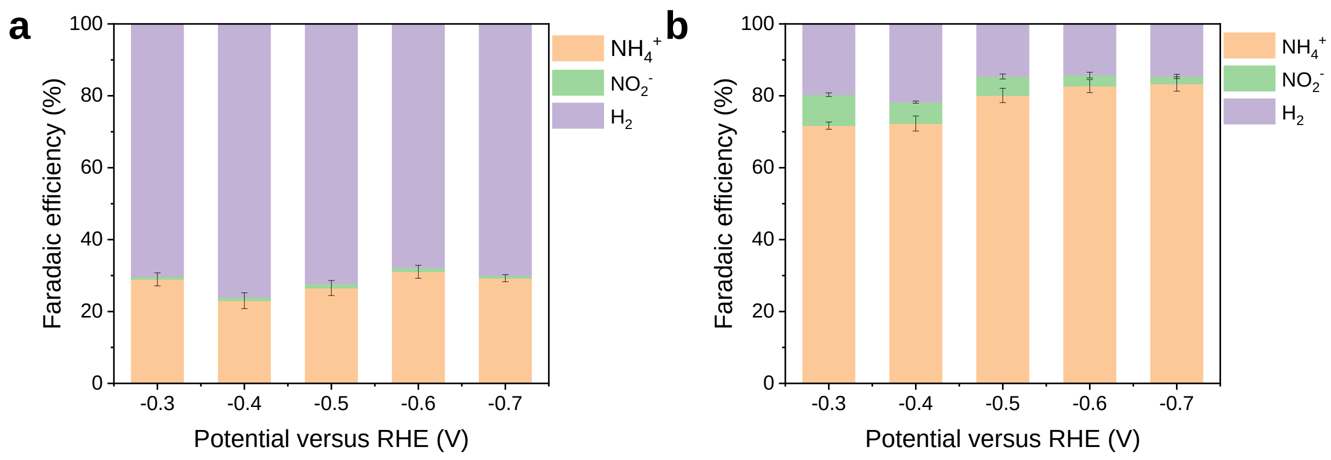

Supplementary Fig.29 | Product distribution and corresponding FE at different potentials. (**a**) TiO_2 and (**b**) Fe-TiO_2. All electrochemical data shown in the figures are not iR-corrected. Source data are provided as a Source Data file.

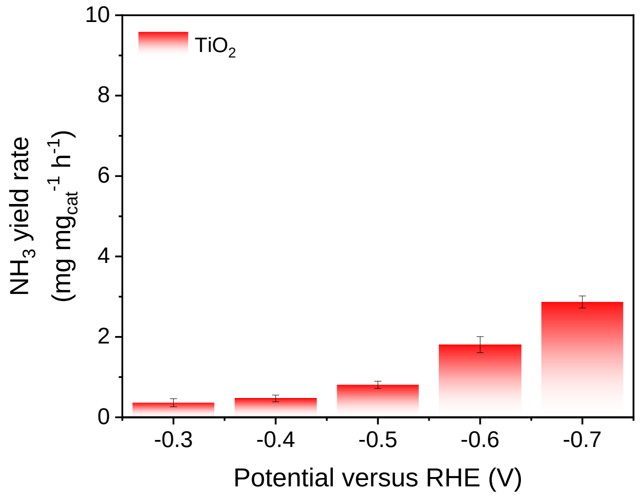

Supplementary Fig.30 | Yield rate of NH_3 for TiO_2 at different potentials. All electrochemical data shown in the figures are not iR-corrected.

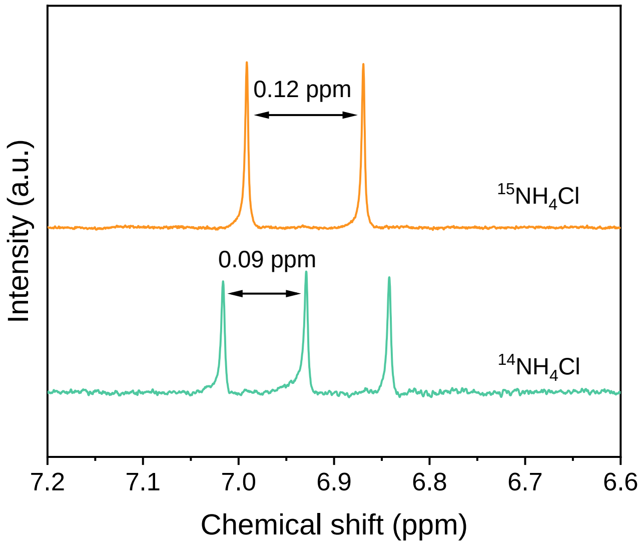

Supplementary Fig. 31 | ^{1}H NMR spectra of $^{14}NH_4Cl$ and $^{15}NH_4Cl$ as NH_4^+ sources. Source data are provided as a Source Data file.

The ^{1}H NMR spectrum of $^{14}NH_4^+$ shows a triplet peak, while the 1H NMR spectrum of $^{15}NH_4^+$ exhibits a doublet peak.

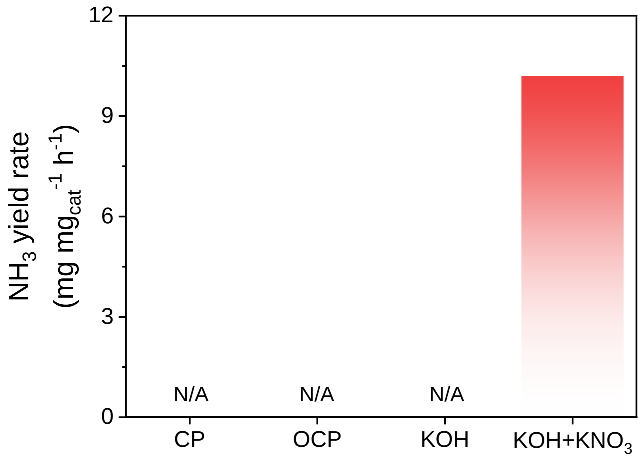

Supplementary Fig. 32 | NH_3 yield rate for carbon paper (CP) at -0.6V vs. RHE; NH_3 yield rate for Fe-TiO_2 without added KNO_3; NH_3 yield rate for Fe-TiO_2 at OCP. Source data are provided as a Source Data file.

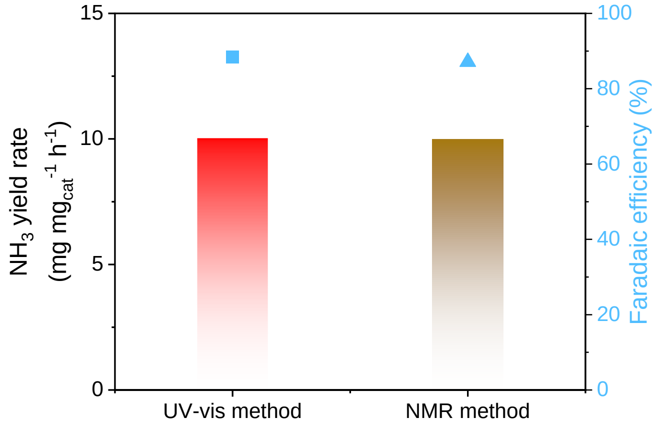

Supplementary Fig. 33 | Quantitative analysis of NH_3 using UV-Vis and NMR methods after chronoamperometry test at -0.6 V vs. RHE for Fe-TiO_2. Source data are provided as a Source Data file.

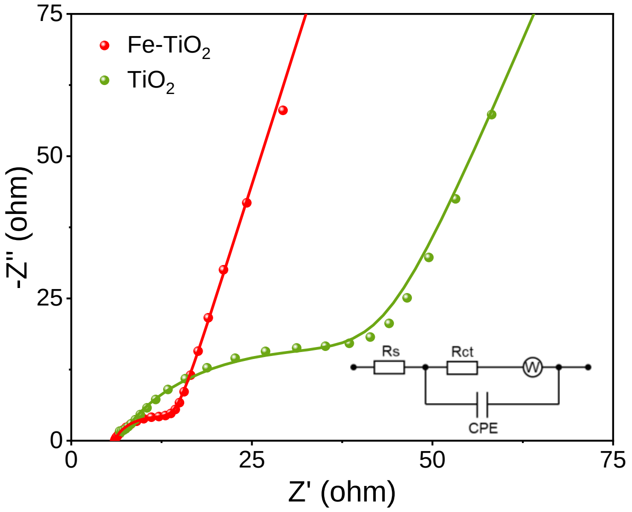

Supplementary Fig. 34 | Electrochemical impedance spectroscopy (EIS) of Fe-TiO_2 and TiO_2. Source data are provided as a Source Data file.

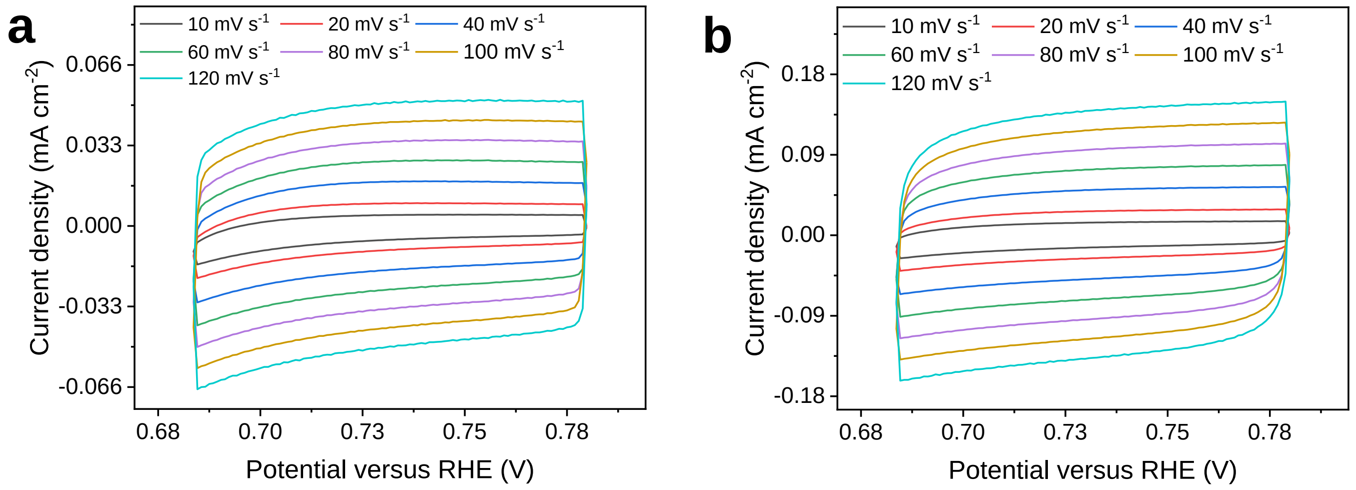

Supplementary Fig. 35 | CV curves of TiO_2 (**a**) and Fe-TiO_2 (**b**) in the non-faradaic region at different scan rates. All electrochemical data shown in the figures are not iR-corrected. Source data are provided as a Source Data file.

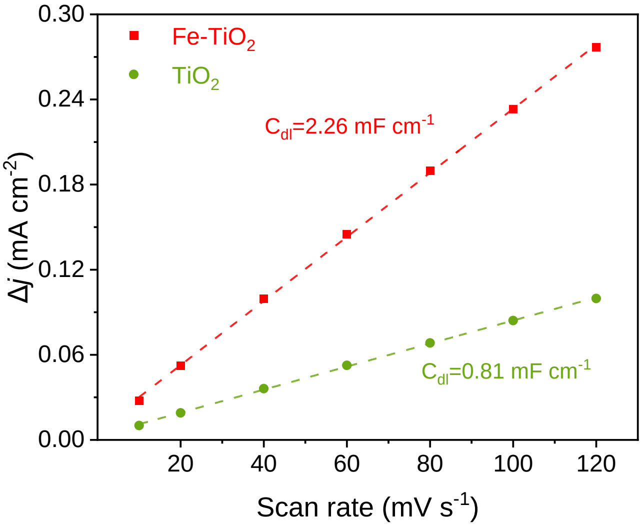

Supplementary Fig. 36 | Calculated ECSA for TiO_2 and Fe-TiO_2 catalysts. Source data are provided as a Source Data file.

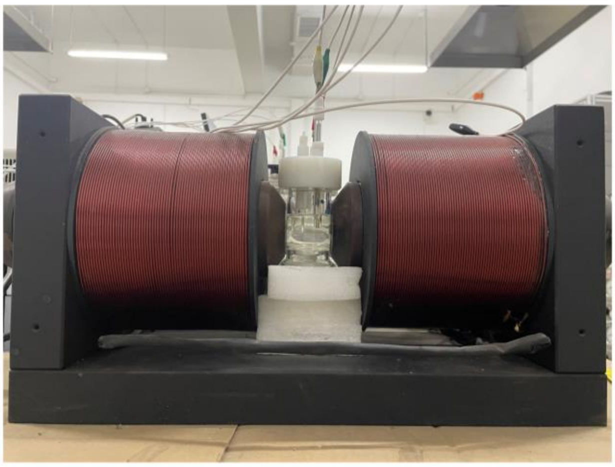

Supplementary Fig. 37 | Experimental setup for electrocatalysis under an external magnetic field.

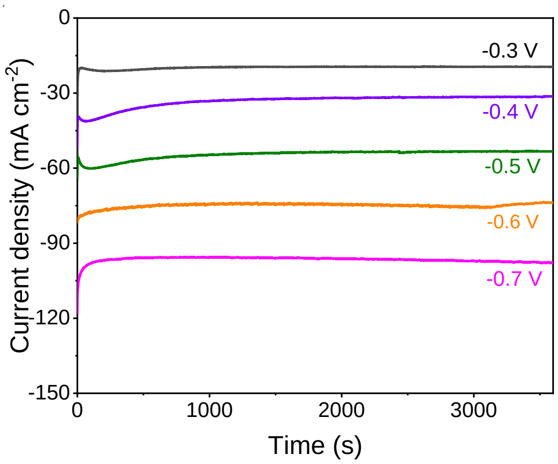

Supplementary Fig. 38 | I-t curve for Fe-TiO_2 at different potentials under an external magnetic field. Source data are provided as a Source Data file.

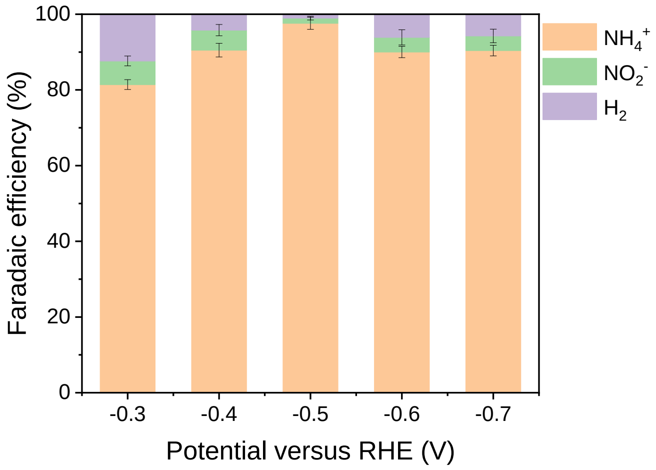

Supplementary Fig. 39 | FE of Fe-TiO_2-M at different potentials under a magnetic field. All electrochemical data shown in the figures are not iR-corrected. Source data are provided as a Source Data file.

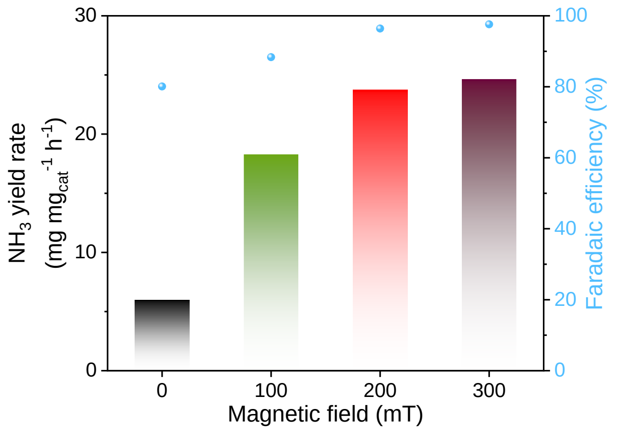

Supplementary Fig. 40 | The NH_3 yield rate and FE at different magnetic fields. Source data are provided as a Source Data file.

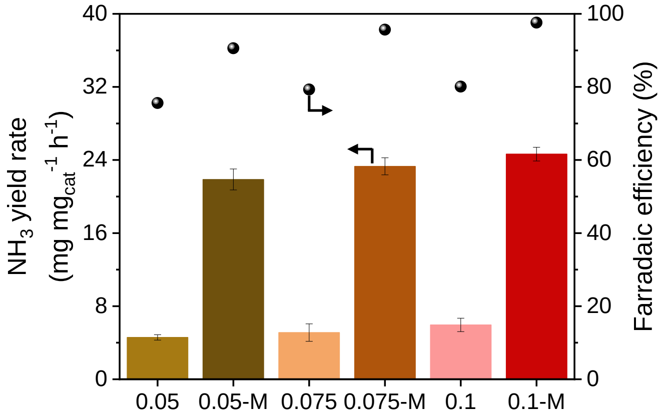

Supplementary Fig. 41 | The NH_3 yield rate and FE at different NO_3^- concentrations (0.05 mol/L,0.075mol/L,0.1mol/L. Source data are provided as a Source Data file.

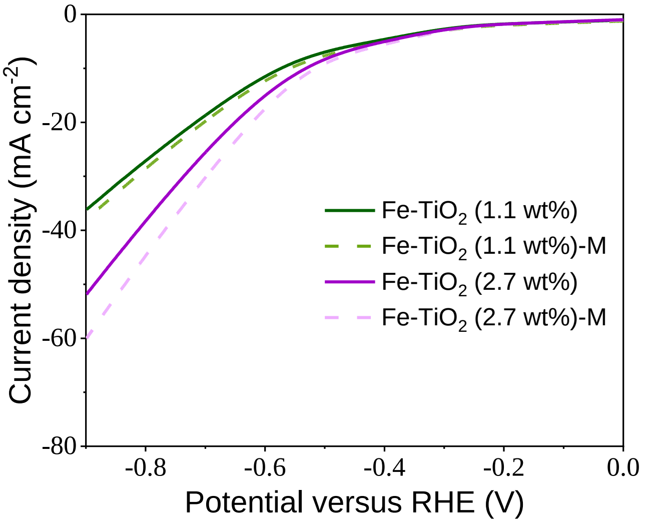

Supplementary Fig. 42 | The LSV curves of Fe-TiO_2 with different loading were obtained with and without the magnetic field opened. The solid line and the dotted line represent with and without the magnetic field opened., respectively. All electrochemical data shown in the figures are not iR-corrected. Source data are provided as a Source Data file.

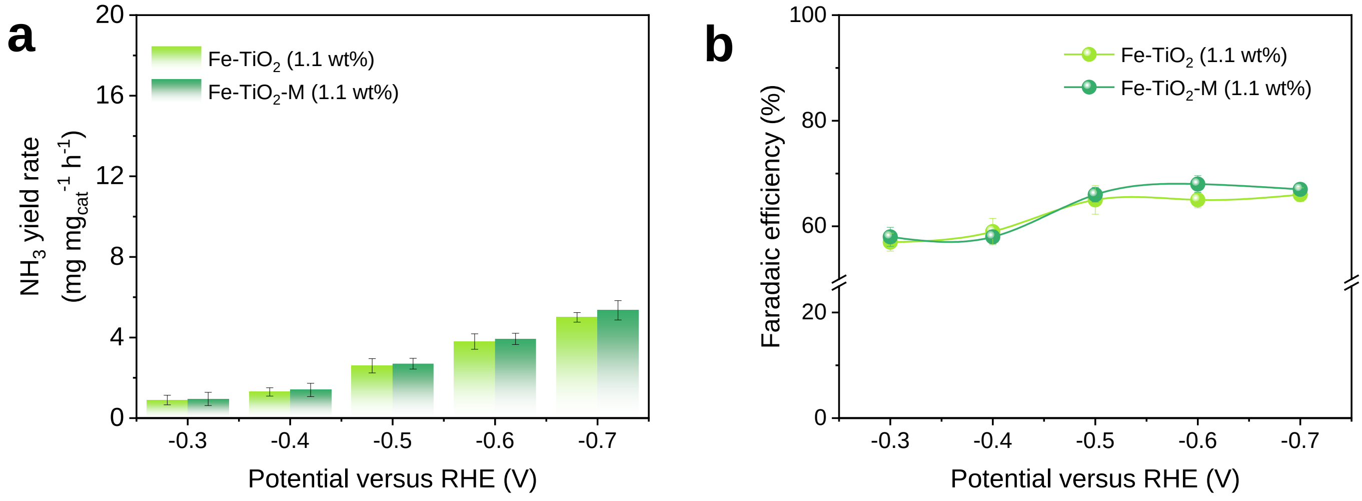

Supplementary Fig. 43 | Diagram of electrochemical testing under a magnetic field. NH_3 yield (**a**) and FE_{NH3} (**b**) of Fe−TiO_2 (1.1 wt%) catalyst at various potentials in the psresence or absence of 300 mT external magnetic fields. All electrochemical data shown in the figures are not iR-corrected. Source data are provided as a Source Data file.

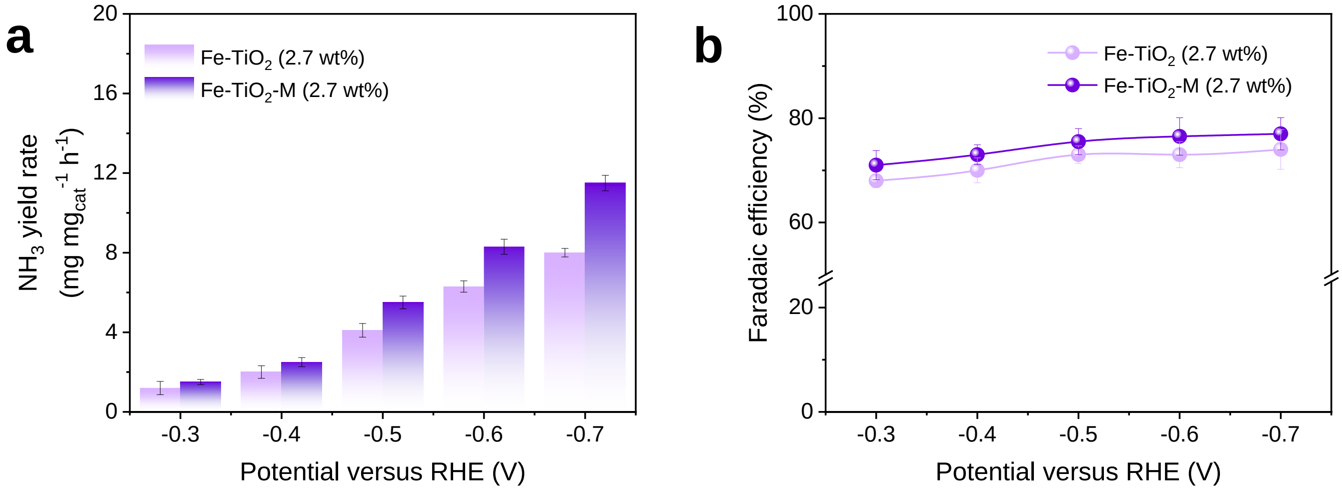

Supplementary Fig. 44 | Diagram of electrochemical testing under a magnetic field. NH_3 yield (**a**) and FE_{NH3} (**b**) of Fe–TiO_2 (2.7 wt%) catalyst at various potentials in the presence or absence of 300 mT external magnetic fields. All electrochemical data shown in the figures are not iR-corrected. Source data are provided as a Source Data file.

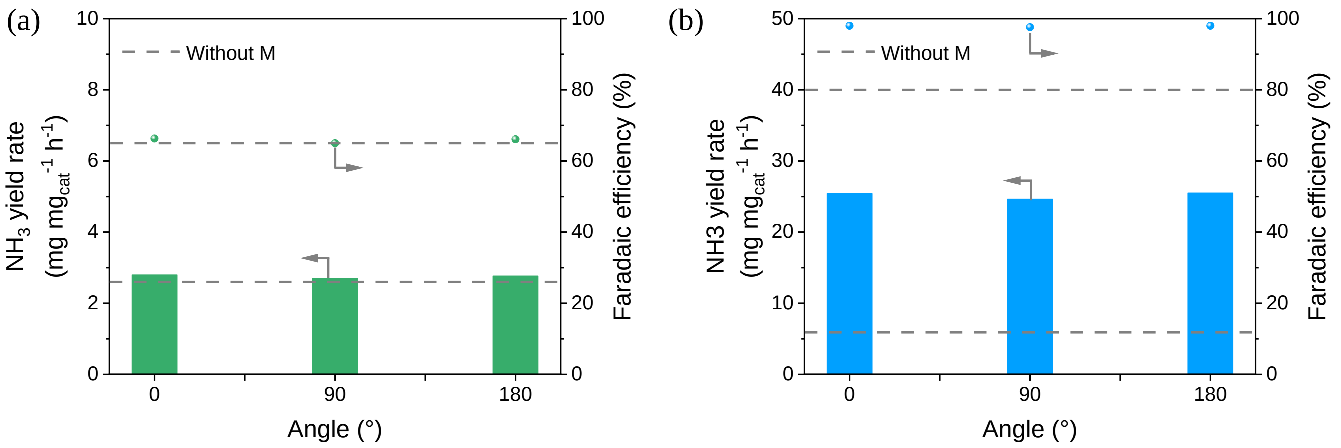

Supplementary Fig. 45| Electrochemical performance under varying magnetic field orientations. Dashed lines represent data in the without magnetic field: (a) 1.1 wt%, (b) 6.8 wt%s Fe-TiO_2. Source data are provided as a Source Data file.

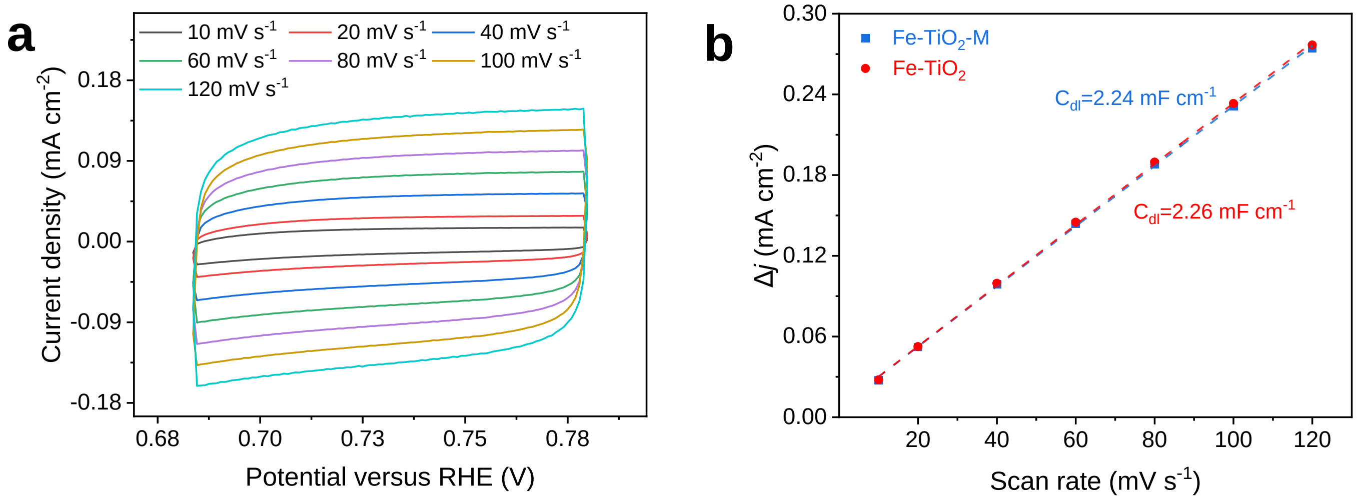

Supplementary Fig. 46 | CV Curves of Fe-TiO_2 in the non-Faradaic region at different scan rates under a magnetic field (**a**), and calculated ECSA (**b**) for Fe-TiO_2 Catalysts (labeled as Fe-TiO_2-M). Source data are provided as a Source Data file.

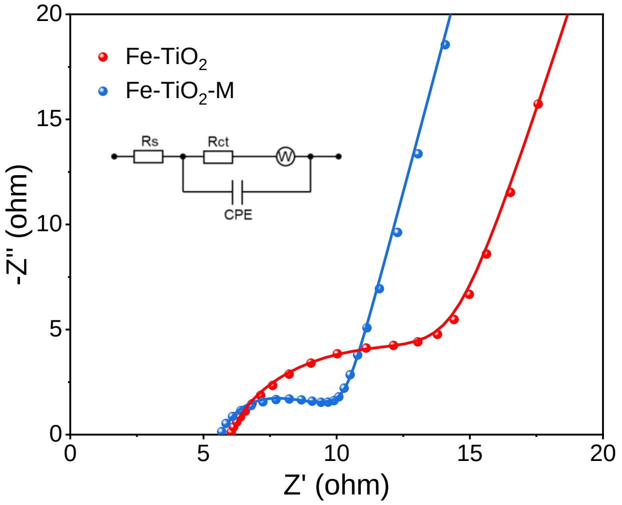

Supplementary Fig. 47 | Electrochemical impedance spectroscopy (EIS) of Fe-TiO_2 under no magnetic field (labeled as Fe-TiO_2) and with an open magnetic field (labeled as Fe-TiO_2-M). Source data are provided as a Source Data file.

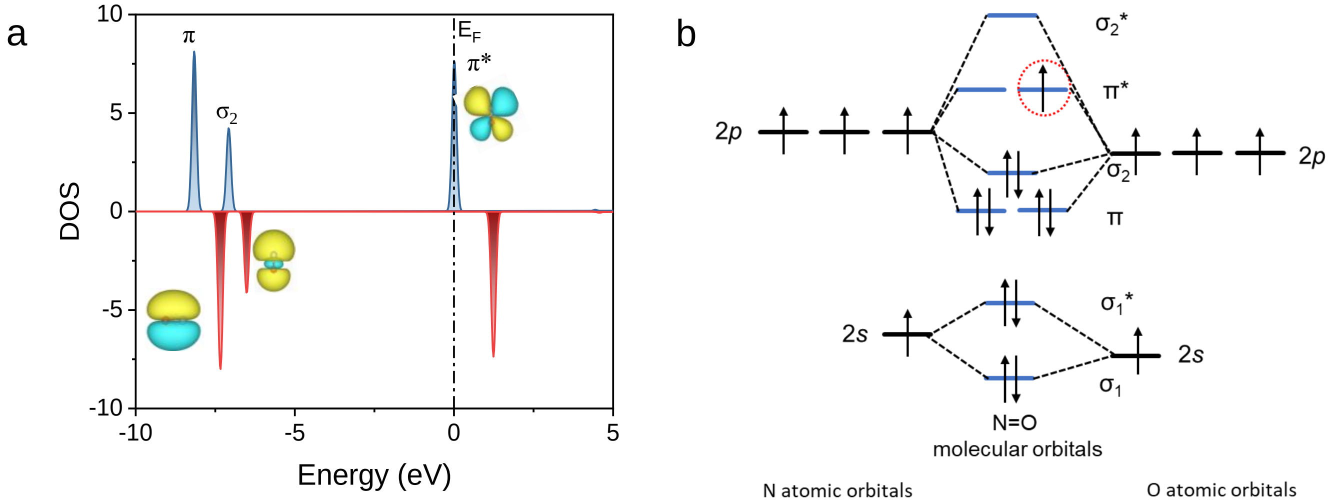

Supplementary Fig. 48 | (**a**) Density of States diagrams for NO. (**b**) NO molecular orbitals. Source data are provided as a Source Data file.

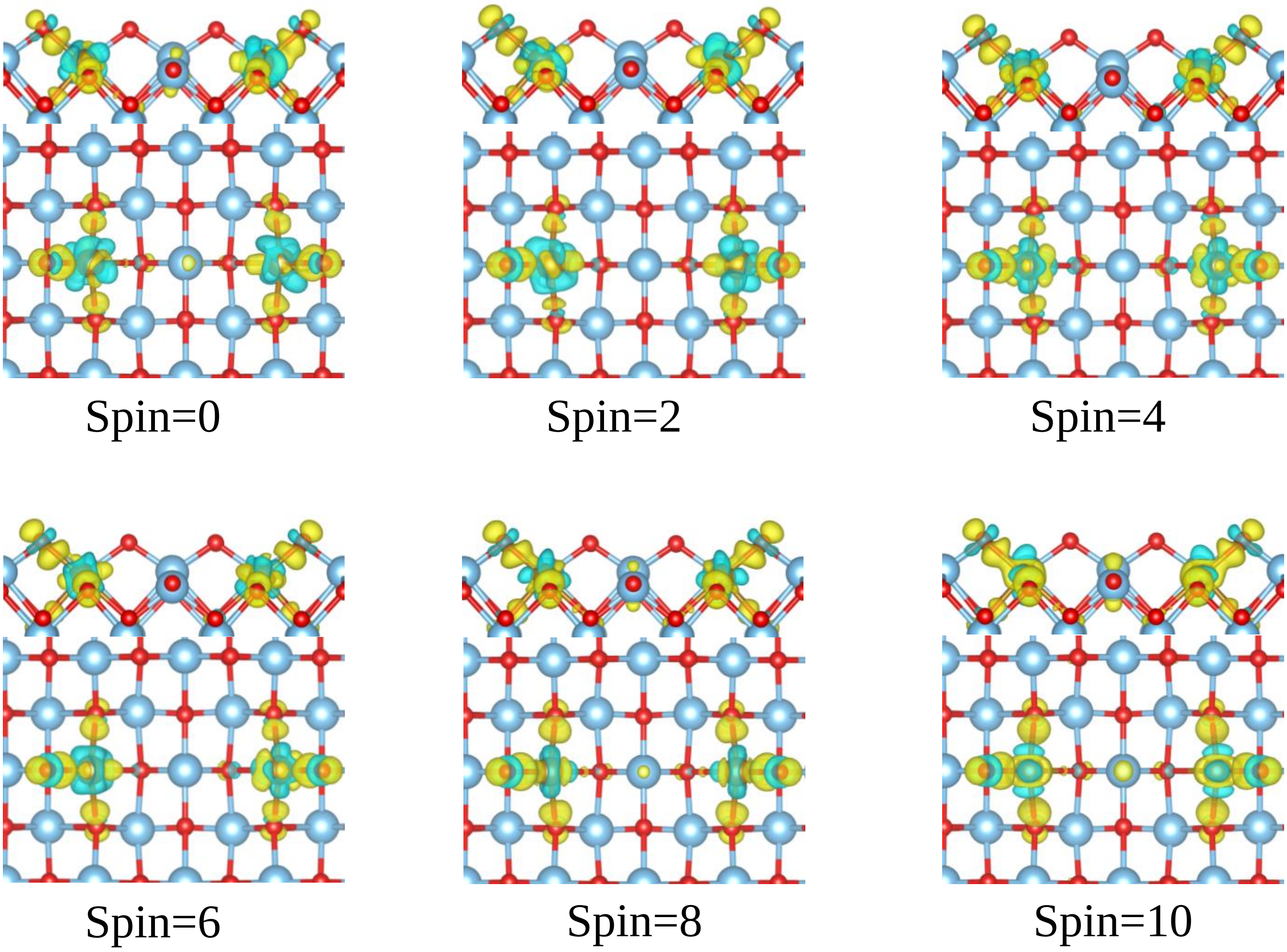

Supplementary Fig. 49 | The charge density difference of different spin configurations Fe-TiO_2. The isosurface value is set to be 0.01 e/$Bohr^3$.

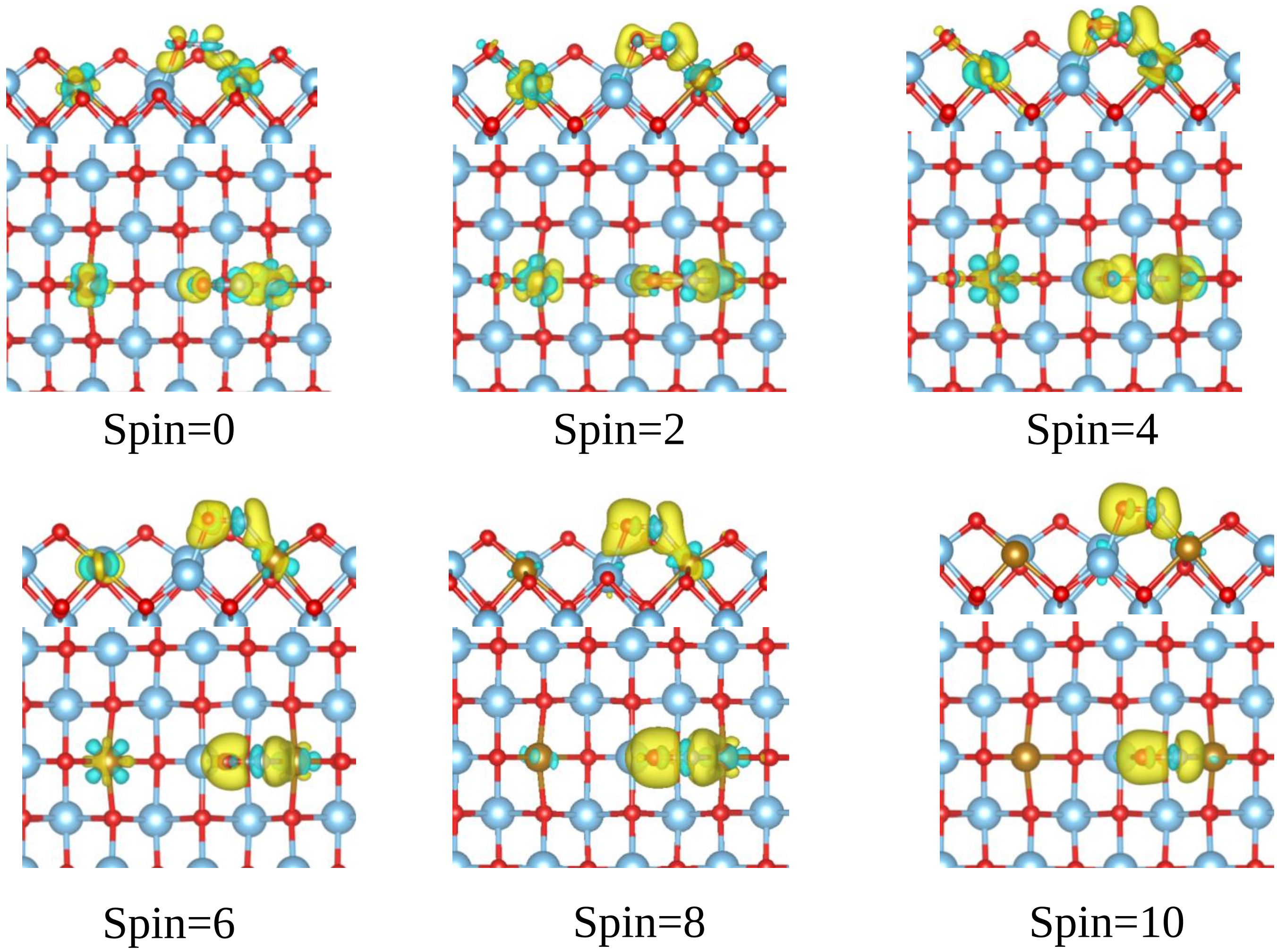

Supplementary Fig. 50 | The charge density difference of different spin configurations NO adsorbed on Fe-TiO_2. The isosurface value is set to be 0.01 e/$Bohr^3$.

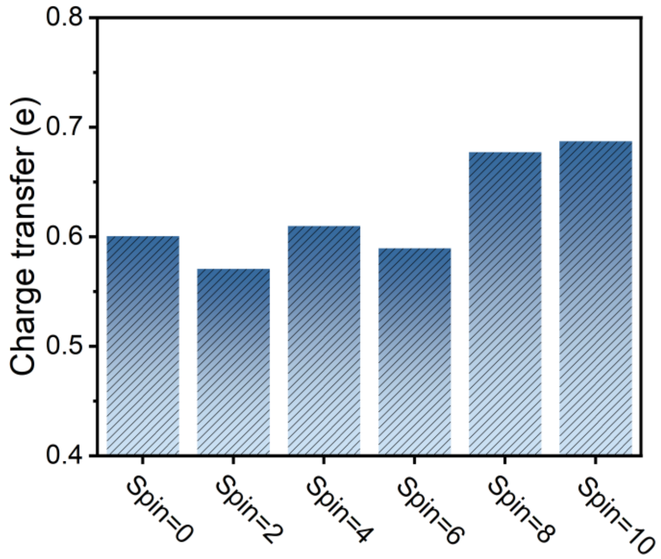

Supplementary Fig. 51 | The charge transfer of different spin configurations NO adsorbed on Fe-TiO_2. Source data are provided as a Source Data file.

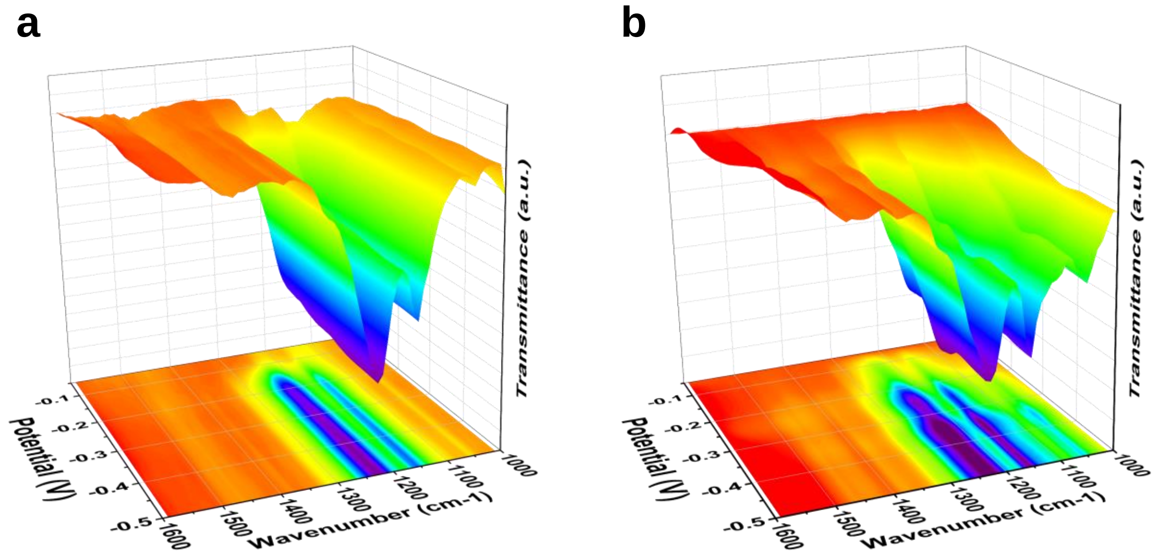

Supplementary Fig. 52 | The 3D *in situ* ATR-FTIR spectra of Fe-TiO_2 with negative scan from OCP to -0.5 V vs. RHE in the absence (**a**) or presence (**b**) of external magnetic fields. All electrochemical data shown in the figures are not iR-corrected.

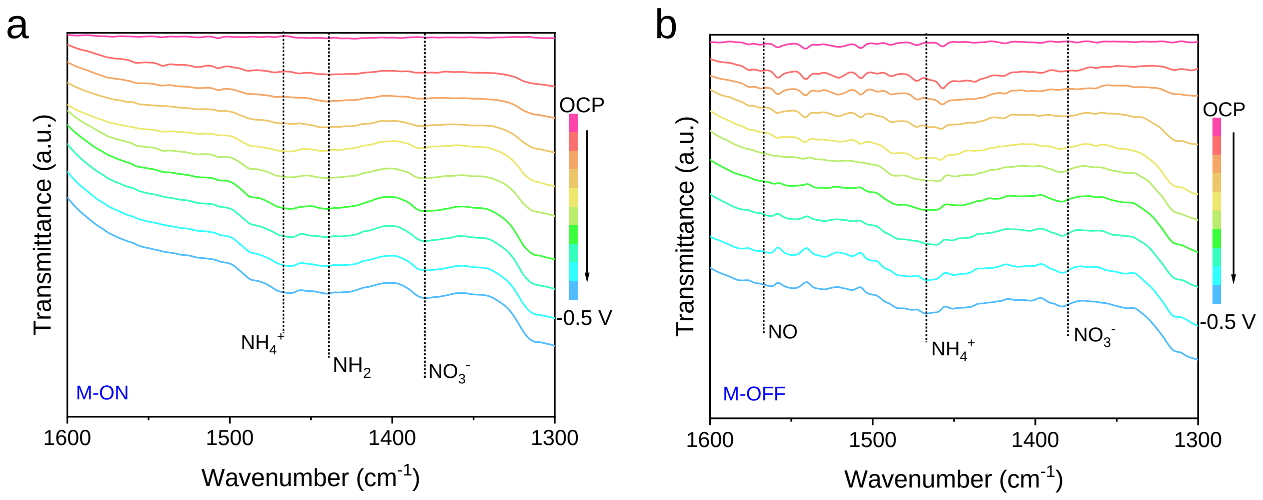

Supplementary Fig. 53 | *In-situ* ATR-FTIR spectra of Fe-TiO_2 with negative scan from OCP to -0.5 V vs. RHE in the presence (**c**) or absence (**d**) of external magnetic fields. All electrochemical data shown in the figures are not iR-corrected. Source data are provided as a Source Data file.

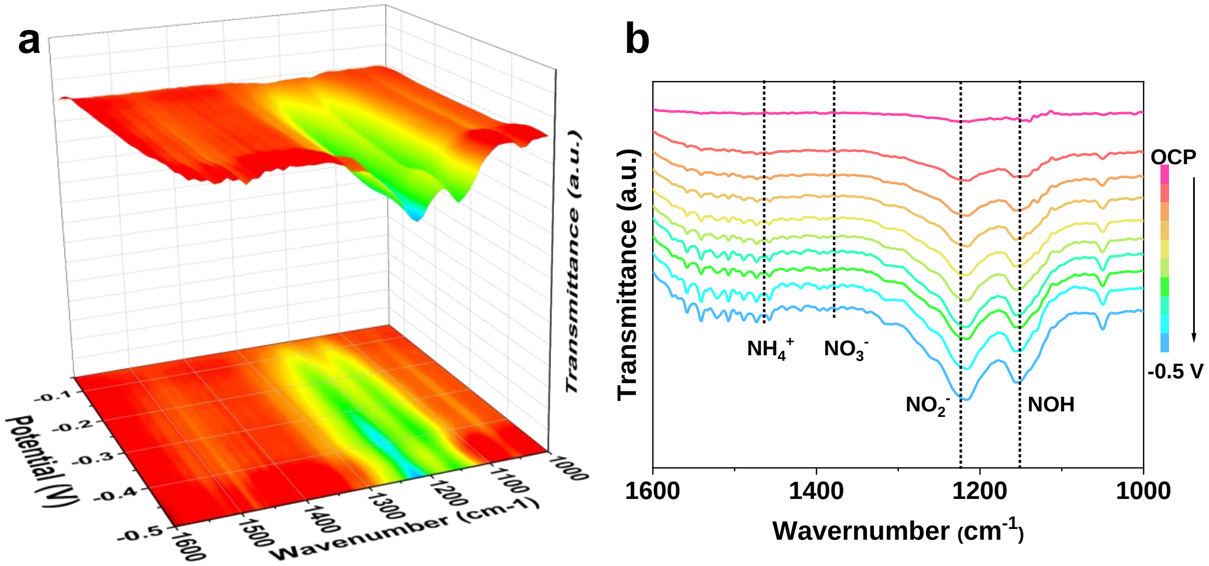

Supplementary Fig. 54 | *In situ* ATR-FTIR Spectra of TiO_2 during Negative Scan from OCP to -0.5 V vs. RHE. All electrochemical data shown in the figures are not iR-corrected. Source data are provided as a Source Data file.

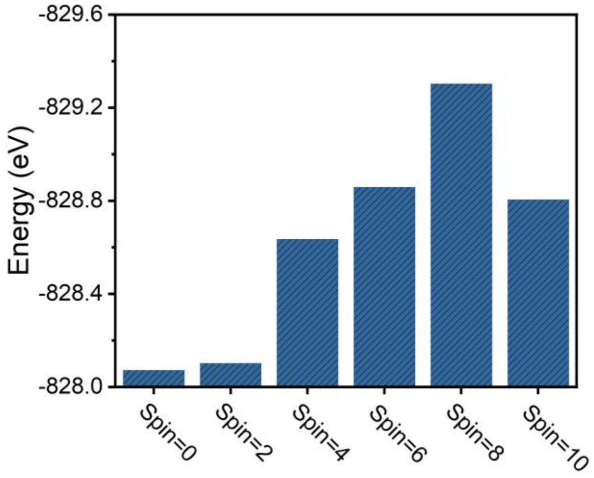

Supplementary Fig. 55| Surface energy for different spin configurations. Source data are provided as a Source Data file.

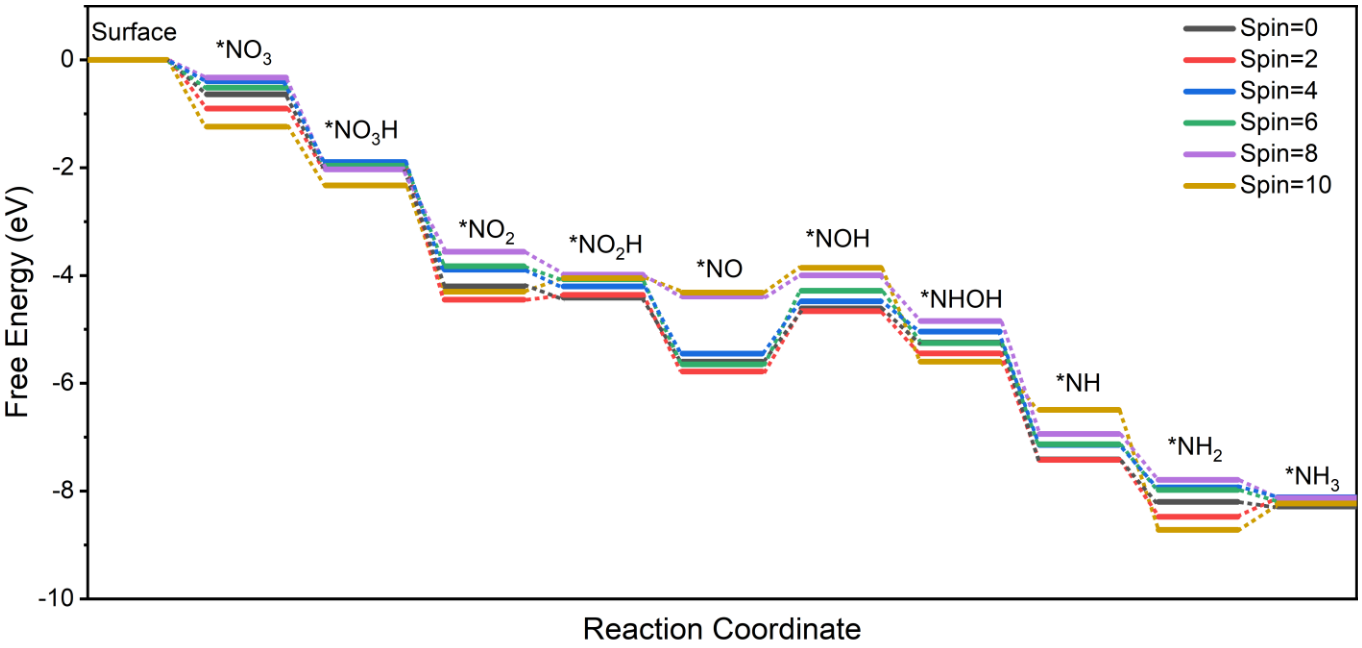

Supplementary Fig. 56 | Free energy diagram for nitrite electroreduction on Fe-TiO_2 under different spin configurations. Source data are provided as a Source Data file.

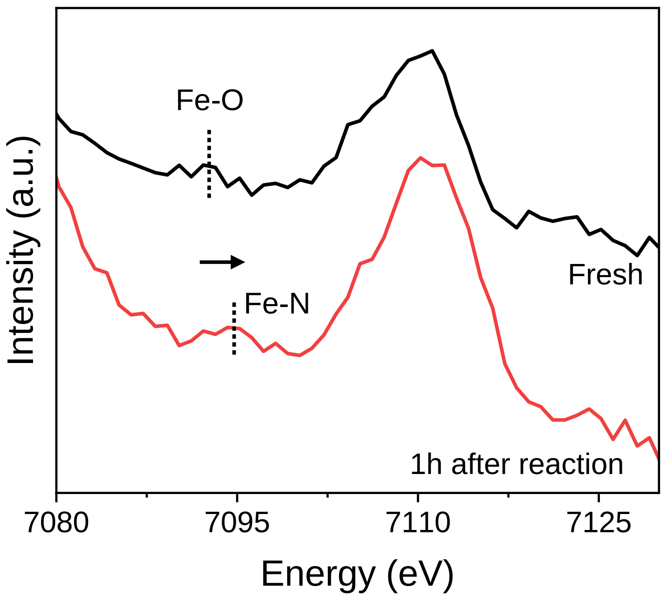

Supplementary Fig. 57 | The X-ray emission spectroscopy of Fe-TiO_2. Source data are provided as a Source Data file.

To experimentally confirm the adsorption of nitrogen atoms on Fe, X-ray emission spectroscopy (XES) was conducted on the catalyst before and after the reaction. At higher emission energies, valence electron transitions to the metal 1s core hole (i.e., the $K\beta_{2,5}$/$K\beta''$ or V2C region) can be observed. These transitions are attributed to ligand np → metal 1s ($K\beta_{2,5}$, ~7110 eV) and ligand ns → metal 1s ($K\beta''$, ~7095 eV) transitions. Therefore, $K\beta''$ is typically used to identify information about different ligands bound to the metal, particularly distinguishing elements such as nitrogen (N), oxygen (O), and carbon (C). As shown in the Supplementary Fig. 57, after 1 hour of reaction, a significant shift in the $K\beta''$ position (from 7092.7 eV to 7094.7 eV) was observed. Based on previous studies [21], this shift is attributed to the adsorption of nitrogen atoms on Fe. Therefore, it is concluded that Fe serves as the adsorption site for N.

Supplementary Table 1. Structural parameters of Fe-TiO_2 extracted from the EXAFS fitting.

Sample	Path	C.N.	R(Å)	$\sigma^2 \times 10^3$ (Å^2)	ΔE	R factor
Fe-TiO_2	Fe-O	5.0±0.4	1.97±0.01	8.8±1.0	-3.1±1.0	0.003
	Fe-Ti	5.0±0.8	2.96±0.01	10±1.7	2.6±1.2	

S_0^2 is the amplitude reduction factor $S_0^2=1$; CN is the coordination number; R is interatomic distance (the bond length between central atoms and surrounding coordination atoms); σ^2 is Debye-Waller factor (a measure of thermal and static disorder in absorber-scatterer distances); ΔE_0 is edge-energy shift (the difference between the zero kinetic energy value of the sample and that of the theoretical model). R factor is used to value the goodness of the fitting

Supplementary Table 2. Catalytic performance of selected NO_3RR catalysts in Fig. 3d.

Catalyst	NO_3^- concentration	Electrolyte	Potential (V vs. RHE)	NH_3 FE (%)	NH_3 production rate (mg h^{-1} mg_{cat}^{-1})	ref.
CuSA NPC	500 mg L^{-1}	0.01 M PBS	-1.1	85.2	5.30	*(2)*
Au-NC/TiO_2	0.05 M	0.2 M Na_2SO_4	-0.6	91	1.92	*(3)*
CuCN/NHCSs	0.1 M	0.1 M NaOH	-1.1	89	21.41	*(4)*
Fe SAC	0.1 M	0.1 M KOH	-0.66	75	5.245	*(5)*
Fe-N/P-C	0.0143 M	0.1 M KOH -	-0.4	82.07	0.95	*(6)*
Cu/Pd/CuOx	50 mg L^{-1} NO_3^--N	0.5M K_2SO_4	-0.643	84.04	1.51	*(7)*
Fe_2Co-MOF	50 mg L^{-1} NO_3^--N	0.05M H_2SO_4	-1.1	90.55	3.46	*(8)*
FeOOH/CP	0.1M	0.1M PBS	-0.8	67	2.42	*(9)*
LF0.9Cu0.1	50 ppm	0.5M Na_2SO_4	-0.9	48	0.35	*(10)*
In-S-G	0.1 M	1M KOH	-0.5	75	3.74	*(11)*
Cu@CuHHTP	0.5 M	0.5M Na_2SO_4	-0.9	67.55	3.68	*(12)*
Fe_2TiO_5	0.1	PBS	-0.9	87.6	12.41	*(13)*
SA-Fe(II)	200PPM-N	0.1 M PBS buffer with 0.5 M Na_2SO_4	-1	99	8.26	*(14)*
Cu/CuxO/GDY	0.1	1 M KOH	-0.8	99.8	25.4	*(15)*
p-$TPTCrCl_3$	0.1	phosphate buffer	-0.75	90	6.12	*(16)*
Fe SAC	0.5	0.1K_2SO_4	−0.66	75	4.5	*(17)*
RuFe NFs	0.1	0.5 Na_2SO_4	-0.6	~80	~15	*(18)*
Fe SAC/NC	0.01	1KOH	-0.8	98.2	22.52	*(19)*
Cu/NC	0.12	0.5Na_2SO_4	-0.7	~100	7.48	*(20)*
Fe-TiO_2	0.1 M	0.1 M KOH	-0.6	97.60	30.15	This work

Supplementary Table 3. Catalytic performance of 1.1wt% Fe-TiO_2.

	NH_3 yield rate (mg mg_{cat}^{-1} h^{-1})			Faraday efficiency (%)		
	Without	With	growth rate	Without	With	growth rate
-0.3 V	0.9	1.0	5.6%	57.0	58.0	1.8%
-0.4 V	1.3	1.4	7.7%	58.0	59.0	1.7%
-0.5 V	2.6	2.7	3.8%	65.0	66.0	1.5%
-0.6 V	3.8	3.9	3.4%	65.0	67.0	3.1%
-0.7 V	5.0	5.4	7.0%	66.0	67.0	1.5%

Supplementary Table 4. Catalytic performance of 2.7wt% Fe-TiO_2.

	NH_3 yield rate (mg mg_{cat}^{-1} h^{-1})			Faraday efficiency (%)		
	Without	With	growth rate	Without	With	growth rate
-0.3 V	1.2	1.5	25.0%	68.0	71.0	4.4%
-0.4 V	2.0	2.5	25.0%	70.0	73.0	4.3%
-0.5 V	4.1	5.5	34.1%	73.0	75.5	3.4%
-0.6 V	6.3	8.3	31.7%	73.0	76.5	4.8%
-0.7 V	8.0	11.5	43.8%	74.0	77.0	4.1%

Supplementary Table 5. Catalytic performance of 6.8wt% Fe-TiO_2.

	NH_3 yield rate (mg mg_{cat}^{-1} h^{-1})			Faraday efficiency (%)		
	Without	With	growth rate	Without	With	growth rate
-0.3 V	1.7	4.2	150.0%	71.7	81.4	13.5%
-0.4 V	3.0	11.9	300.0%	72.3	90.5	25.2%
-0.5 V	5.9	24.6	314.3%	80.1	97.6	21.9%
-0.6 V	10.2	30.2	195.6%	82.7	90.0	8.9%
-0.7 V	16.2	34.8	114.7%	83.3	90.4	8.5%

Supplementary Table 6. Catalytic performance under varying magnetic field orientations of 1.1 wt% Fe-TiO_2.

	NH_3 yield rate (mg mg_{cat}^{-1} h^{-1})			Faraday efficiency (%)		
	Without	With	growth rate	Without	With	growth rate
0	2.6	2.8	7.7%	65	66.3	2.0%
90	2.6	2.7	3.8%	65	65.2	0.3%
180	2.6	2.77	6.5%	65	66.1	1.7%

Supplementary Table 7. Catalytic performance under varying magnetic field orientations of 6.8 wt% Fe-TiO_2.

	NH_3 yield rate (mg mg_{cat}^{-1} h^{-1})			Faraday efficiency (%)		
	Without	With	growth rate	Without	With	growth rate
0	5.9	25.4	330.5%	80	98	22.5%
90	5.9	24.6	316.9%	80	97.6	22.0%
180	5.9	25.5	332.2%	80	98	22.5%

Supplementary Table 8. Electrochemical impedance spectroscopy (EIS) fitting data.

	Rs (Ω)	Rct (Ω)
TiO_2	6.712	33.02
Fe-TiO_2	6.074	7.557
Fe-TiO_2-M	5.855	2.664

The fitting analysis of Nyquist plots reveals that the charge transfer resistance (Rct) is reduced from 33.02 Ω to 7.56 Ω by Fe doping, indirectly suggesting that Fe serves as an active site for NO_3RR. Furthermore, the fitting of Nyquist plots obtained under an external magnetic field shows a further reduction of Rct from 7.56 Ω to 2.66 Ω, indicating that the electron transfer rate in the reaction is significantly enhanced by the magnetic field.

Supplementary Reference

1. Pan, L. et al. Single-atom or dual-atom in TiO_2 nanosheet: Which is the better choice for electrocatalytic urea synthesis? *Angew. Chem. Int. Edit.* **135**, e202216835 (2023).

2. Zhao, X. et al. Boosting the selectivity and efficiency of nitrate reduction to ammonia with a single-atom Cu electrocatalyst. *Chem. Eng. J.* **466**, 143314 (2023).

3. Yang, M. et al. Au nanoclusters anchored on TiO_2 nanosheets for high-efficiency electroreduction of nitrate to ammonia. *Nano Res.* **17**, 1209-1216 (2024).

4. Liu, Y. et al. Unlocking the potential of sub-nanometer-scale copper via confinement engineering: a remarkable approach for electrochemical nitrate-to-ammonia conversion in wastewater treatment. *Chem. Eng. J.* **475**, 146176 (2023).

5. Wu, Z. Y. et al. Electrochemical ammonia synthesis via nitrate reduction on Fe single atom catalyst. *Nat. Commun.* **12**, 2870 (2021).

6. Xu, J. et al. Breaking local charge symmetry of iron single atoms for efficient electrocatalytic nitrate reduction to ammonia. *Angew. Chem. Int. Edit.* **62**, e202308044 (2023).

7. Ren, T. et al. Interfacial polarization in metal-organic framework reconstructed Cu/Pd/CuOx multi-phase heterostructures for electrocatalytic nitrate reduction to ammonia. *Appl. Catal. B-Environ. Energy* **318**, 121805 (2022).

8. Lv, Y. et al. Highly efficient electrochemical nitrate reduction to ammonia in strong acid conditions with Fe_2M-trinuclear-cluster metal-organic frameworks. *Angew. Chem. Int. Edit.* **62**, e202305246 (2023).

9. Liu, Q. et al. High-performance electrochemical nitrate reduction to ammonia under ambient conditions using a FeOOH nanorod catalyst. *ACS Appl. Mater. Interfaces* **14**, 17312-17318 (2022).

10. Chu, K. et al. Cation substitution strategy for developing perovskite oxide with rich oxygen vacancy-mediated charge redistribution enables highly efficient nitrate electroreduction to ammonia. *J. Am. Chem. Soc.* **145**, 21387-21396 (2023).

11. Lei, F. et al. Electrochemical synthesis of ammonia by nitrate reduction on indium incorporated in sulfur doped graphene. *Chem. Eng. J.* **426**, 131317 (2021).

12. Zhu, X. et al. Filling mesopores of conductive metal-organic frameworks with Cu clusters for selective nitrate reduction to ammonia[J]. *ACS Appl. Mater. Interfaces* **14**, 32176-32182 (2022).

13. Du, H. et al. Durable electrocatalytic reduction of nitrate to ammonia over defective pseudobrookite Fe_2TiO_5 nanofibers with abundant oxygen vacancies. *Angew. Chem. Int. Edit.* **135**, e202215782 (2023).

14. Liu, C. et al. Specifically adsorbed ferrous ions modulate interfacial affinity for high-rate ammonia electrosynthesis from nitrate in neutral media. *Proc. Natl. Acad. Sci. U.S.A.* 120, e2209979120 (2023).

15. Feng, X. et al. Cu/Cu_xO/Graphdiyne tandem catalyst for efficient electrocatalytic nitrate reduction to ammonia. *Adv. Mater.* 2405660 (2024).

16. Askari, M. J. et al. Selective Reduction of Aqueous Nitrate to Ammonium with an Electropolymerized Chromium Molecular Catalyst. *J. Am. Chem. Soc.* **146**, 7439-7455 (2024).

17. Wu, Z. Y. et al. Electrochemical ammonia synthesis via nitrate reduction on Fe single atom catalyst. *Nat. Commun.* 12, 2870 (2021).

18. Wang, Y. et al. Atomic coordination environment engineering of bimetallic alloy nanostructures for efficient ammonia electrosynthesis from nitrate. *Proc. Natl. Acad. Sci. U.S.A.* **120**, e2306461120 (2023).

19. Cheng, X. et al. Unveiling structural evolution of Fe single atom catalyst in nitrate reduction for enhanced electrocatalytic ammonia synthesis. *Nano Res.* 1-7 (2024).

20. Yin, H. et al. Unraveling the Activity Trends and Design Principles of Single-Atom Catalysts for Nitrate Electrocatalytic Reduction. *ACS Nano* **17**, 25614-25624 (2023).
21. Lancaster K. M. et al. X-ray emission spectroscopy evidences a central carbon in the nitrogenase iron-molybdenum cofactor. Science 334, 974-977 (2011).
