# Supplementary material for: Ferromagnetic Fe-TiO2 spin catalysts for enhanced ammonia electrosynthesis
Source: Nat Commun. 2025 Jan 28;16:1129. doi: 10.1038/s41467-025-56566-7 (PMC11775347; doi:10.1038/s41467-025-56566-7)
Supplement: Supplementary file 2 — Description of Additional Supplementary Files [file 41467_2025_56566_MOESM2_ESM.pdf]

DESCRIPTION OF ADDITIONAL SUPPLEMENTARY FILES

Supplementary Data 1 - Supplementary Data 1 contains the atomic coordinates of the optimized computational model used in this work
